# The Caspase-Activated DNase drives inflammation and contributes to defense against viral infection

**DOI:** 10.1038/s41418-024-01320-7

**Published:** 2024-06-07

**Authors:** Abdul Moeed, Nico Thilmany, Frederic Beck, Bhagya K. Puthussery, Noemi Ortmann, Aladin Haimovici, M. Tarek Badr, Elham Bavafaye Haghighi, Melanie Boerries, Rupert Öllinger, Roland Rad, Susanne Kirschnek, Ian E. Gentle, Sainitin Donakonda, Philipp P. Petric, Jonas F. Hummel, Elisabeth Pfaffendorf, Paola Zanetta, Christoph Schell, Martin Schwemmle, Arnim Weber, Georg Häcker

**Affiliations:** 1 Institute of Medical Microbiology and Hygiene, Medical Center, University of Freiburg, Faculty of Medicine, Freiburg, Germany; 2 Institute of Medical Bioinformatics and Systems Medicine, Medical Center - University of Freiburg, Faculty of Medicine, University of Freiburg, Freiburg, Germany; 3 German Cancer Consortium (DKTK) and German Cancer Research Center (DKFZ), partner site Freiburg, Freiburg, Germany; 4 Institute of Molecular Oncology and Functional Genomics, Department of Medicine II and TranslaTUM Cancer Center; TUM School of Medicine, Technical University of Munich, Munich, Germany; 5 Institute of Molecular Immunology and Experimental Oncology, TUM School of Medicine, Technical University of Munich, Munich, Germany; 6 Institute of Virology, Medical Center, University of Freiburg, Faculty of Medicine, Freiburg, Germany; 7 Institute of Surgical Pathology, Medical Center, University of Freiburg, Faculty of Medicine, Freiburg, Germany; 8 BIOSS Centre for Biological Signalling Studies, University of Freiburg, Freiburg, Germany

**Keywords:** Cell death and immune response, Infectious diseases

## Abstract

Mitochondria react to infection with sub-lethal signals in the apoptosis pathway. Mitochondrial signals can be inflammatory but mechanisms are only partially understood. We show that activation of the caspase-activated DNase (CAD) mediates mitochondrial pro-inflammatory functions and substantially contributes to host defense against viral infection. In cells lacking CAD, the pro-inflammatory activity of sub-lethal signals was reduced. Experimental activation of CAD caused transient DNA-damage and a pronounced DNA damage response, involving major kinase signaling pathways, NF-κB and cGAS/STING, driving the production of interferon, cytokines/chemokines and attracting neutrophils. The transcriptional response to CAD-activation was reminiscent of the reaction to microbial infection. CAD-deficient cells had a diminished response to viral infection. Influenza virus infected CAD-deficient mice displayed reduced inflammation in lung tissue, higher viral titers and increased weight loss. Thus, CAD links the mitochondrial apoptosis system and cell death caspases to host defense. CAD-driven DNA damage is a physiological element of the inflammatory response to infection.

## Introduction

Mitochondria can act in host defense through the decision to trigger apoptosis in response to noxious stimuli. More recently, it has become clear that mitochondria can also release various factors that drive inflammation: mitochondrial (mt) DNA, double-stranded (ds) RNA and formylated peptides as well as inflammasome-activating molecules can be released and trigger an inflammatory response [1]. The release of these molecules may occur through vesicular transport [2] or through apoptotic permeabilization of outer and inner mitochondrial membranes [3, 4].

While apoptosis causes the release of these pro-inflammatory factors, apoptotic cell death is non-inflammatory because cell death caspases, which are invariably activated during apoptosis, suppress these inflammatory signals, and experimental blockade of caspase-activity is required to unmask the inflammatory activity [5–7]. However, the discovery of sub-lethal mitochondrial signaling has added an important aspect: the mitochondrial apoptosis pathway can also be active at only low intensity and in the absence of cell death. Although caspases are activated and some downstream events can be measured [8], the caspase activity appears to be too low to counteract the inflammatory signals [9]. Remarkably, all tested bacterial and viral infections triggered sub-lethal mitochondrial signals that led to the secretion of cytokines and chemokines from infected cells [9, 10], suggesting a role of these signals in host defense. mtDNA [9] and the mitochondrial intermembrane space protein SMAC [10] have been implicated in sub-lethal pro-inflammatory signaling. However, the signals downstream of mitochondria are only partially understood.

It is intriguing to note that sub-lethal signals activate the caspase-activated DNase (CAD). CAD is a well-characterized nuclease known to cleave genomic DNA during apoptosis [11, 12]. CAD is however not necessary for apoptosis in cell culture or in mice, nor is it essential for apoptotic DNA-degradation in vivo [13]. The apoptosis-associated activation of CAD therefore appears to be dispensable, suggesting that CAD-dependent cleavage of genomic DNA has a physiological function outside cell death. One reported function of CAD is in the differentiation of skeletal muscle progenitor cells, where CAD-dependent cleavage in its promoter has been linked to the up-regulation of p21 [14]. In cancer cells, CAD contributes to tumor growth [15], is necessary to evade the consequences of genotoxic stress [16] and can generate chromosomal misalignments and micronuclei [17]. CAD-induced DNA-damage triggers a DNA-damage response (DDR), most easily detectable by the histone phosphorylation mark γH2AX [8].

DNA-damage induced by chemicals or by irradiation and the subsequent DDR cause the secretion of cytokines and chemokines through major inflammatory signaling pathways [18–21]. The findings that first, DNA-damage and the DDR are pro-inflammatory and secondly, DNA-damage is caused by sub-lethal signaling during infection, suggest the possibility that a CAD-dependent DDR, resulting from sub-lethal mitochondrial signaling during infection, may contribute to inflammation and immune response. We tested this hypothesis and report a significant contribution of CAD-activity to host defense. Experimental activation of CAD triggered a complex pro-inflammatory response through the major signaling pathways of innate immunity. Loss of CAD impaired the secretion of pro-inflammatory mediators and the immune response during viral infection. The results suggest that CAD-induced DNA-damage is an integral part of the initial response of the mammalian organism to microbial infection, and that the immune system uses the CAD-dependent DDR as an alert signal.

## Results

### CAD contributes to the secretion of inflammatory mediators during sub-lethal signaling

**Fig. 1:**
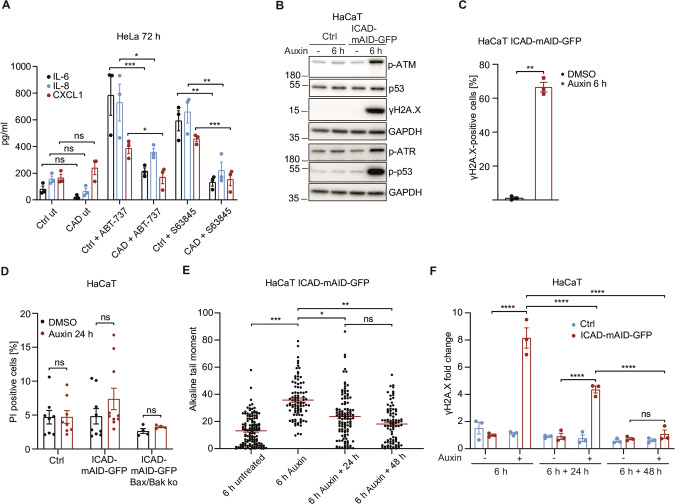
CAD contributes to cytokine induction through sub-lethal signals, and activation of CAD causes transient, non-lethal DNA-damage and DDR. **A** HeLa cells (control or deficient in CAD) were treated with the BCL-2/BCL-X_L_/BCL-w-inhibitor ABT-737 (10 µM) or the Mcl-1 inhibitor S63845 (100 nM) for 72 h. IL-6, IL-8 and CXCL-1 concentrations were determined in the supernatants. Data means/SEM of at least three experiments. Significance was determined by two-way ANOVA, Sidak’s post-hoc test. **B** Western blots showing induction of DDR-phosphorylation events after 6 h auxin (20 µM) treatment of HaCaT ICAD-mAID-GFP cells or control cells (Ctrl). The blot shows one of three very similar results. **C** Quantification of γH2AX-positive cells after 6 h auxin-treatment (20 µM) as detected by immunofluorescence microscopy. Three separate experiments were performed and analyzed. For each sample, 5 random pictures were taken and a minimum of 80 cells were quantified. Graph shows the mean value/SEM of the three experiments. Statistical analysis was done with a paired two-sample *t*-test. **D** HaCaT cells were treated with solvent (DMSO) or auxin (20 µM) for 24 h. Cells were stained with propidium iodide (PI) and analyzed by flow cytometry. Shown are means/SEM of at least 4 experiments. Significance was tested by two-way ANOVA, Sidak’s post-hoc test. ns, *p* ≥ 0.05. **E** Alkaline comet assay to assess DNA-damage. HaCaTICAD-mAID-GFP cells were treated for 6 h with auxin (20 µM) and analyzed either directly or after 24 or 48 h following auxin wash-out. Each symbol represents one cell. Data are from three separate experiments, and at least 70 nuclei were assessed per condition. Statistical analysis was done using nested one-way ANOVA with Sidak’s multiple comparisons test. Tail moment = tail DNA [%] x tail length. **F** Time course of γH2AX-positivity after CAD activation in HaCaT (Control) and HaCaT ICAD-mAID-GFP cells under the conditions described in (**E**). Signals were quantified from Western blots and are shown as means/SEM of *n* = 3 independent experiments. Significance was determined by two-way ANOVA, Sidak’s post-hoc test. For all samples: ns, *p* ≥ 0.05, *p < 0.05; **p < 0.01; ***p < 0.001; ****p < 0.0001.

While apoptosis is non-inflammatory, sub-lethal signals in the mitochondrial apoptosis pathway can lead to the secretion of IL-6 and of chemokines (IL-8, CXCL1) [9]. We used sub-lethal concentrations of BCL-2-family inhibitors to trigger mitochondrial signals and to test for a contribution from CAD. As reported before [9], ABT-737 (inhibitor of BCL-2/BCL-X_L_/BCL-w) and S63845 (MCL-1-inhibitor) induced the secretion of IL-6, IL-8 and CXCL1 from HeLa cells. We found that this secretion was strongly reduced when the cells lacked CAD (Fig. 1A; deletion efficiency for all cells is shown in Fig. S1). There was no difference in cell number between treated control and CAD-deficient cells (Fig. S2A) although some cell death occurred especially during the first 48 h of treatment (Fig. S2B); IL-6 production was mostly seen in the time period between 48 and 72 h (Fig. S2C). To test whether dead cells contributed to factor production by stimulating surviving cells, we removed dead cells at 48 h or not (i.e., took off non-adherent cells and added them back or not) and measured IL-6 and chemokine levels at 72 h (Fig. S2D–G). We observed a small effect on chemokine but not IL-6 secretion when ‘dead’ (non-adherent) cells treated with 50 nM (but not 100 nM) of S63845 were added back (Fig. S2F, G). While it is difficult to exclude residual chemokine secretion from transferred cells, at least the majority of secreted factors appears not to be produced in response to stimulation by dead cells.

We had previously reported that STING is required for IL-6-secretion upon treatment with BH3-mimetics in this system [9]. While we saw no clear CAD-dependent STING-phosphorylation as a measure of its activation (although it may be difficult to catch the right time point), cGAS-dependent IRF3-phosphorylation was detectable, and this was reduced in CAD-deficient cells (Fig. S2H, I). Although cGAS and CAD may act in parallel in different pathways, the data are compatible with the interpretation that cGAS/STING-activation occurs downstream of CAD. We investigated this axis further below.

CAD-activity is therefore required for the full cellular inflammatory response to sub-lethal signals in the mitochondrial apoptosis pathway. It has been reported that sub-lethal signals triggered by ABT-737 cause a CAD-dependent γH2AX-response [8] (see also Fig. S2H,J). One way of interpreting this is that CAD triggers a DDR with an inflammatory component. We went on to test this hypothesis.

### Activation of CAD causes transient, non-lethal DNA-damage and a DDR

To measure a CAD-dependent DDR, we used a system where CAD can be activated directly. Prior to activation, CAD is inactive in a complex with its inhibitor, ICAD. CAD is activated when cell death caspases cleave and inactivate ICAD. We have generated a cellular model to activate CAD caspase-independently, by direct proteasomal degradation of ICAD [17]. In these cells, the endogenous CAD-inhibitor ICAD has been replaced with the fusion construct ICAD-mAID-GFP, expressed together with the rice F-box protein Tir1 [22]. In the presence of the plant hormone auxin, the ICAD-mAID-GFP-construct is ubiquitylated and degraded, causing the activation of CAD (Fig. S3A) [23]. The construct was expressed in HaCaT human keratinocytes [17] and in HeLa cells. A DDR (measured by the histone phosphorylation signal, γH2AX) was detectable within the first hour of auxin-treatment, and degradation of ICAD proceeded over about one day (Fig. S3B-E). We observed prominent activation of major DDR kinases, such as ATM and ATR, as well as phosphorylation of p53 and H2AX (Fig. 1B, S3F). There was little change in cell cycle distribution of HeLa cells stimulated for 6 h with auxin, and slightly more cells were in G2/M phase 24 h after stimulation (Fig. S3G). When we stained for γH2AX-positive cells after 6 h of auxin-stimulation, the positive population had more G0/G1 cells than the γH2AX-negative population indicating a slightly higher response of cells in this cycle phase (Fig. S3H). The presence of N-acetyl cysteine did not appreciably alter the γH2AX-signal suggesting that the response was independent of reactive oxygen species (Fig. S3I). After 6 h, about 60-80% of the cells showed signs of the DDR (positive nuclear staining for γH2AX, Fig. 1C, S4A-C) while cell death after 24 h was around 5% (Fig. 1D, S4D), indicating that the large majority of cells survived the activation of CAD. By microscopy, the γH2AX-stain appeared almost homogenous throughout the nucleus, suggesting the occurrence of many individual DNA-strand breaks. The cell death induced was blocked by co-deletion of BAX and BAK in the auxin-inducible HaCaT cells (Fig. 1D), suggesting that some cells died as a consequence of apoptosis triggered by the DNA-damage. Actual DNA-damage in the cells could be measured after 6 h of auxin-treatment by alkaline Comet assay; when auxin was washed out after 6 h, DNA-damage was repaired over about 48 h (Fig. 1E), and the DDR subsided with similar kinetics (Fig. 1F, S5A–E). Auxin-stimulation of HaCaT ICAD-mAID-GFP cells caused the appearance of 53BP1-positive foci and also (although fewer) RAD51-positive foci, which had mostly disappeared 24 h after auxin-washout (Fig. S5F, G). This suggests that both non-homologous end joining (53BP1) and homologous recombination (RAD51) are involved in the repair processes following non-lethal CAD-activation.

It can be difficult to distinguish between single- (SSB) and double-strand DNA breaks (DSB). The alkaline Comet assay (Fig. 1E) is assumed to detect both SSB and DSB while the neutral Comet assay is positive only in the case of DSB [24], and CAD can in principle induce both forms of strand breaks [11, 16]. Auxin-dependent CAD-activation also caused a positive signal in the neutral Comet assay (Fig. S3J), indicating that CAD induced DSB (and presumably also SSB). The data indicate transient, non-lethal DNA-damage and DDR upon the activation of CAD.

### CAD induces a pro-inflammatory DNA-damage response

**Fig. 2:**
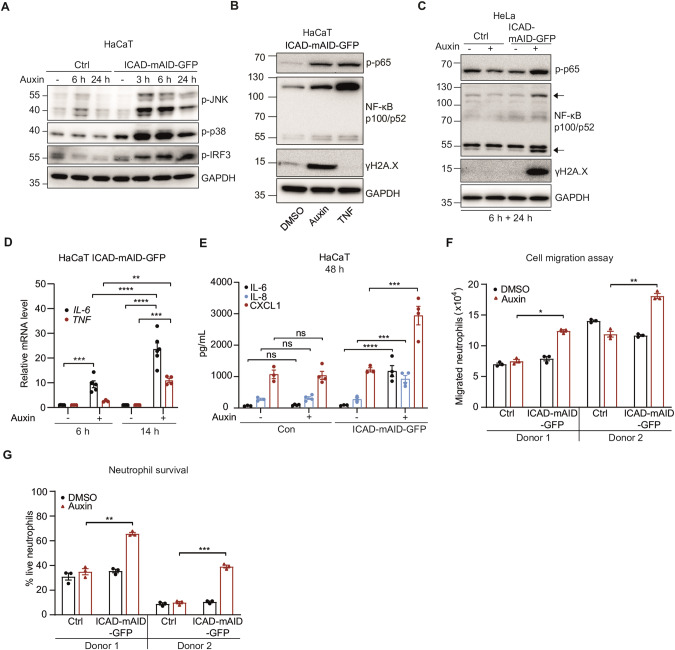
CAD triggers a pro-inflammatory DNA-damage response. **A** HaCaT cells (control or ICAD-mAID-GFP) were treated with solvent or auxin (20 µM) for 24 h. Cells lysates were analyzed by Western Blot. Blots are representative of 3–5 experiments. **B** HaCaT ICAD-mAID-GFP cells were treated with solvent or auxin for 6 h. Auxin was washed out and culture was continued for 24 h. As a positive control for p65-phosphorylation, cells were treated with TNF (100 ng). Cells were harvested and analyzed by Western Blot for p-p65 and p100/p52 (representative of three separate experiments). **C** HeLa cells (control or ICAD-mAID-GFP cells) were treated with solvent (DMSO (-)) or auxin (5 µM) for 6 h. Auxin was washed out and culture was continued for 24 h. Cell lysates were analyzed by Western blotting for NF-κB p-p65 and p100/p52. A blot representative of three independent experiments is shown. **D** RT-qPCR analyses of *TNF and IL-6* mRNA levels in HaCaT ICAD-mAID-GFP cells after auxin (20 µM) treatment. Data represents means/SEM of at least four independent experiments (each symbol shows one experiment). Data were normalized to GAPDH and then to the DMSO control (-). Statistical significance was tested by two-way ANOVA, Tukey’s post-hoc test. **E** HaCaT cells were treated with solvent or auxin (20 µM) for 6 h. Auxin was washed out and culture was continued for 48 h. Supernatants were collected and IL-6, IL-8 and CXCL-1 were measured by ELISA. Shown are means/SEM of at least three experiments. Significance was determined by two-way ANOVA, Sidak’s post-hoc test. **F**, **G** HeLa control cells or HeLa ICAD-mAID-GFP cells were treated with auxin (5 µM) or DMSO for 6 hours, then auxin was washed out and culture was continued for 48 hours. Cell culture supernatants (1:2 (donor 1 or 1:5 (donor 2) dilutions) were used in transwell assays with human neutrophils isolated from two healthy donors to measure chemotaxis (**F**) or neutrophil survival by AnnexinV/PI staining (**G**). Data are means/SEM of three independently derived supernatants. Significance was tested by two-way ANOVA, Sidak’s multiple comparisons test. For all samples: ns, *p* ≥ 0.05, *p < 0.05; **p < 0.01; ***p < 0.001; ****p < 0.0001.

Activation of CAD led to the activation of the major pro-inflammatory signaling pathways, as indicated by the phosphorylation of the kinases JNK, p38 and ERK (ERK was activated only in HeLa cells), phosphorylation of IRF3 (Fig. 2A, S6A) and activation of canonical NF-κB (RelA/p65) (Fig. 2B, C). We also observed activation of NF-κB2/p100 (alternative NF-κB) in HeLa cells (Fig. 2C) and the induction of NF-κB promoter-activity upon CAD-activation (Fig. S6B). Auxin-treatment for 6 or 14 h induced transcription of *TNF* and *IL-6* (Fig. 2D), and the cells secreted high amounts of IL-6, CXCL1 and IL-8 over a period of 48 h (Fig. 2E, S6C). Supernatants from HeLa cells expressing active CAD induced the migration of human neutrophils and inhibited their spontaneous apoptosis (Fig. 2F, G). IL-8 and CXCL1 (whose secretion we had observed upon CAD-activation) are known as potent chemo-attractants towards neutrophils [25]; IL-6 (identified as upregulated both at the protein and mRNA level), IL-1, TNF and GM-CSF (found to be transcriptionally induced by CAD-activation, see below) have all been shown to prolong the half-life of neutrophils [26]; these factors are likely to contribute to this neutrophil-stimulating effect.

DNA-damage induced by the chemical etoposide has previously been found to induce two phases of NF-κB-dependent signals in HeLa cells, the first requiring ATM-activity, the second depending on the activity of the kinase RIPK1 [21]. Cytokine/chemokine secretion upon CAD-activation however was mostly ATM-independent in HaCaT cells: ATM-inhibition had no effect on the levels of IL-8 or CXCL1 and even enhanced secretion of IL-6 (Fig. S6D); in HeLa cells, ATM-inhibition had no significant effect on IL-6 secretion but reduced IL-8 and CXCL-1-levels upon CAD-activation (Fig. S6E). We also deleted ATM and ATR alone or together in the auxin-responsive HeLa cells (the deletion was not quite complete, Fig. S1). Both deletions reduced the response in terms of secretion of the three factors tested; co-deletion of ATM and ATR caused a stronger reduction in the levels of especially IL-8 and CXCL1 (Fig. S6I). Both ATM and ATR therefore appear to be involved in the inflammatory DDR to CAD-induced DNA-damage in HeLa cells.

Conversely, RIPK1-inhibition reduced CXCL1 levels in HaCaT but not HeLa cells and had no effect on the secretion of IL-6 or IL-8 in either cell line (Fig. S6E, F). Inhibition of NF-κB-activation using an IKK-inhibitor reduced the levels of IL-8 and CXCL1 but not IL-6 in HeLa cells (Fig. S6G); in HaCaT cells this inhibitor on its own (in the absence of CAD-activation) increased the secretion of these factors, and no inhibition of CAD-dependent secretion was observed (not shown). P38-kinase inhibition substantially reduced the secretion of all three soluble factors in HaCaT cells (Fig. S6H) but reduced only IL-6 in HeLa cells (Fig. S6G). Thus, the production of soluble inflammatory mediators upon CAD-activation receives contributions from a number of major signaling pathways and shows cell-type variation.

### CAD-activity triggers the cGAS-STING-axis

The cyclic GMP-AMP synthase (cGAS) acts in recognizing accessible dsDNA. Upon DNA-recognition, cGAS synthesizes cGAMP, a ligand for the activation of the stimulator of interferon genes (STING), and STING drives the expression of interferon (IFN) and other inflammatory mediators [27]. Although initially described for the recognition of viral DNA, it is clear that cGAS-STING can be activated by self-DNA that has become accessible. Significantly, cGAS can be activated by genomic DNA following DNA-damage [28–30]. We therefore tested whether CAD could lead to the activation of cGAS and a subsequent STING-response.

**Fig. 3:**
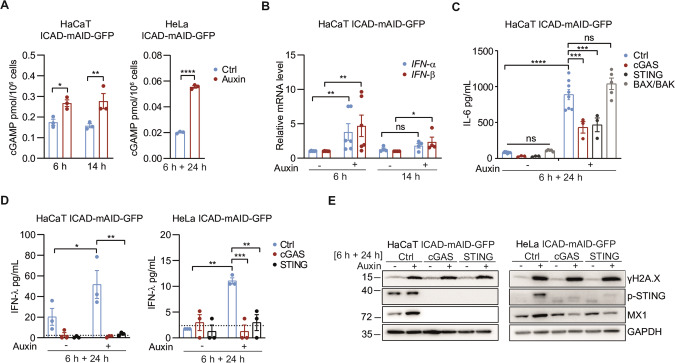
CAD triggers activation of cGAS/STING. **A** cGAMP production by cells expressing active CAD. HaCaT or HeLa ICAD-mAID-GFP cells were treated with solvent (DMSO) or auxin (20 µM (HaCaT) or 5 µM (HeLa)) to activate CAD for the indicated times (6 + 24 h, stimulation for 6 h followed by washing and analysis after 24 h). Total cell lysates were analyzed for cGAMP by ELISA. Data shown are mean/SEM of *n* = 3. All p-values were calculated by two-way ANOVA, Sidak’s post-hoc test (left) and unpaired *t*-test (right). Detection limit was 0.01-0.02 pmol/10^6^ cells. The experiments were also performed with cGAS-deficient HaCaT ICAD-mAID-GFP cells, in which cGAMP upon CAD-activation was consistently under the detection limit (three separate experiments) (**B**) Induction of IFN I-genes by CAD-activation. *IFN-α* and *IFN-β* mRNA was measured by RT-PCR in HaCaT ICAD-mAID-GFP cells after the indicated times of auxin (20 µM) treatment. Data represent means/SEM of at least four independent experiments. Data were normalized to GAPDH and then to the DMSO control (-). Statistical significance was tested using Mann–Whitney test. **C** cGAS/STING contribute to IL-6 secretion from cells expressing active CAD. Supernatants from HaCaT ICAD-mAID-GFP cells (control or cGAS, STING or Bax/Bak deficient) were treated with DMSO or auxin (20 µM) for 6 hours, then auxin was washed out and culture was continued for 24 hours. IL-6 levels were measured by ELISA. **D** Secretion of IFN-λ upon CAD-activation. HeLa and HaCaT ICAD-mAID-GFP cells were treated as in (**C**). IFN-λ concentration was measured by ELISA. Dotted lines show the detection limit. Data shown are means/SEM of three independent experiments. Statistical analysis was performed using two-way ANOVA with Tukey’s test for multiple comparison correction. **E** CAD induces cGAS-dependent STING-phosphorylation and cGAS and STING dependent MX1-expression. HaCaT or HeLa ICAD-mAID-GFP cells and corresponding cells with cGAS or STING deletions were treated with solvent (DMSO (-)) or auxin (HaCaT: 20 µM, HeLa: 5 µM) for 6 h. Auxin was washed out and culture was continued for 24 h (6 h + 24 h). Cells were lysed, and the indicated proteins were analyzed by Western Blot. Shown is a representative Western blot from three independent experiments. Data are means/SEM of at least three experiments. Significance was determined by two-way ANOVA, Tukey´s post-hoc test. For all samples: ns, *p* ≥ 0.05, *p < 0.05; **p < 0.01; ***p < 0.001; ****p < 0.0001.

Activation of CAD increased the levels of the cGAS product and STING-ligand, cGAMP, indicating cGAS-activation (Fig. 3A). CAD-activation induced the transient transcription of *IFN-α* and *IFN-β* genes (Fig. 3B). The induction of IL-6 partly depends on STING-activity [31], and deletion of either cGAS or STING in the cells carrying the ICAD-mAID-GFP-construct reduced IL-6-secretion in response to CAD-activation (Fig. 3C). The secretion of IFN type III (IFN-λ) was detectable in the supernatant upon CAD-activation, and loss of cGAS or STING abolished the secretion of IFN-λ (Fig. 3D). Phosphorylation of STING, a proxy of its activation, was observed upon activation of CAD, and CAD-activation induced the IFN-response gene MX1; both events were not observed in cells lacking cGAS or STING (Fig. 3E). We considered the possibility that CAD-triggered DNA-damage may induce the release of mtDNA through the induction of DNA-damage-dependent apoptosis (or sub-lethal mitochondrial signals). However, deletion of BAX and BAK, which prevented even the little cell death seen upon CAD-activation (Fig. 1D) had no effect on IL-6-secretion, making a major contribution of mtDNA as a cGAS-ligand in this situation unlikely (Fig. 3C). These results show that CAD-activation drives the activation of cGAS/STING and STING-dependent gene induction.

### CAD-activation causes the gene-expression landscape of an anti-infectious response

**Fig. 4:**
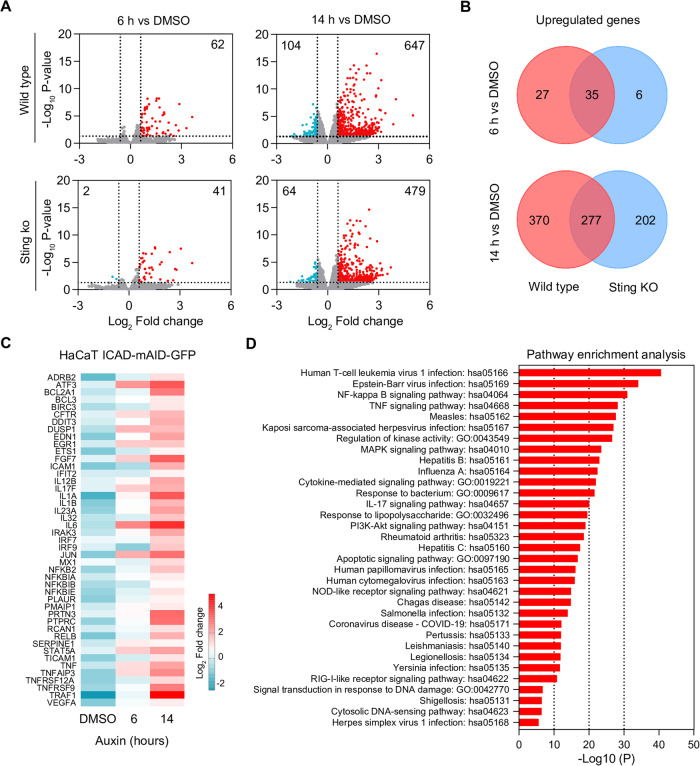
Global RNA expression pattern and pro-inflammatory signaling pathways upon CAD activation in HaCaT-ICAD cells. HaCaT ICAD-mAID-GFP cells and STING-deficient HaCaT ICAD-mAID-GFP cells were stimulated with auxin or with solvent for 6 h or 14 h and subjected to RNA-sequencing. **A** Volcano plot of differentially expressed genes in HaCaT ICAD-mAID-GFP cells showing significantly upregulated and downregulated genes at 6 h (6 h auxin vs. solvent) and14 h (14 h auxin vs. solvent) of stimulation (top two panels). Bottom panels, the same analysis for HaCaT ICAD-mAID-GFP cells lacking STING (14 h). **B** Significantly upregulated genes that are unique to HaCaT ICAD-mAID-GFP cells or STING-deficient HaCaT ICAD-mAID-GFP cells or shared between both genotypes. Top, gene expression after 6 h, bottom after 14 h of stimulation. **C** Heatmap diagram of log_2_ fold change values of gene expression at different time points with respect to their solvent controls after 6 and 14 h of CAD activation. Shown are significantly differentially regulated innate immunity genes (InnateDB database) following protein-protein-interaction network analysis. DMSO, comparison of DMSO 14 vs. 6 h. **D** Pathway enrichment analysis of genes represented in protein-protein-interaction networks (Fig. S5) following CAD activation. **A**–**D**, Genes with adjusted p-values < 0.05 and log_2_ fold change value <-0.6 or >0.6 are considered as significantly differentially expressed. P value < 0.05 was adjusted to consider only statistically significant pathways.

We performed RNA-sequencing of HaCaT ICAD-mAID-GFP cells and found a pronounced transcriptional response to the activation of CAD. There were relatively small changes in gene expression after 6 h of stimulation but substantial gene induction at 14 h of auxin-treatment. We identified 647 significantly upregulated genes after 14 hours of CAD activation (62 genes at 6 h; Fig. 4A). To measure the role of cGAS/STING in gene induction, we conducted the analysis in parallel with STING-deficient ICAD-mAID-GFP cells. About 25% fewer genes were induced by CAD-activation in STING-deficient cells compared to STING-competent cells (Fig. 4A). A number of genes were induced in both cell lines, and a number of genes only in wt and not in STING-deficient cells; perhaps surprisingly, a substantial number of genes were found upregulated only in the absence of STING (Fig. 4B). This would perhaps most easily explained by an inhibitory effect of STING-dependent negative regulatory factors. Pathway enrichment analysis of the genes that were upregulated only in the absence of STING identified a number of different cellular responses without any obvious unified physiological function (Fig. S7A). It is noteworthy that some of the immediately cGAS/STING-dependent responses we determined (cGAMP-production, IFN-synthesis) peaked earlier than the global induction of transcription, suggesting a complex network of signals in the CAD-dependent DDR. However, IFN I was only detected at the mRNA-level and protein may be around longer, and IFN-λ, which signals in a way similar to IFN I, was easily detectable at 24 h after auxin-washout (Fig. 3D).

We then analyzed the genes induced by CAD-activation in wt cells. Among the significantly up-regulated genes were numerous mediators of inflammation and immune regulation, including cytokines, chemokines and signaling mediators (Fig. 4C). We tested for functional interactions of these up-regulated genes to identify underlying signaling pathways in the context of protein-protein interactions (PPIs). Using the STRING database, we generated a network of 936 interactions between 179 proteins containing 37 additional interactomes (Fig. S7B). We further performed functional and topological analysis of PPIs network to identify hub proteins/core interactomes with higher degree of interaction and betweenness centrality within the network (Fig. S7C, D). The cytokines TNF and IL-6 were among the most prominent CAD-induced hub-proteins; p53, although itself not regulated transcriptionally, was identified as a further key interaction hub of the CAD-dependent DDR (Fig. S7C, D). We performed pathway enrichment analysis of these interactomes [32]. Strikingly, this analysis identified the enrichment of numerous pathways of host response to infection with viruses, bacteria and parasites in cells expressing active CAD, as well as major cytokine and pattern recognition receptor signaling pathways (Fig. 4D). Genes from pathways that are known to be induced upon infection with very different pathogens were up-regulated with high significance, from responses to DNA viruses (such as Herpes Simplex virus [HSV], hepatitis B virus, Epstein-Barr virus, human papilloma virus) and RNA viruses (influenza virus, hepatitis C virus, measles virus) to responses to bacteria (*Legionella*, *Yersinia, Shigella*) and parasites (*Leishmania*, *Trypanosoma*). We also found a striking interaction-network of significantly regulated IFN-induced genes (Fig. S7D, E). Pathway enrichment analysis of these IFN-regulated interactomes identified numerous pathways of host response to infection with viruses (Fig. S7F). Previous results had shown that similarly diverse pathogens can all activate CAD through sub-lethal mitochondrial signals [9, 10]. Thus, CAD-activation induced the transcription of genes that are known to be upregulated in response to infection. Because CAD is activated by infection, this suggests that the known response to infection may in fact partly be mediated by CAD-activity.

### CAD-contributes to the secretion of inflammatory mediators during viral infection

**Fig. 5:**
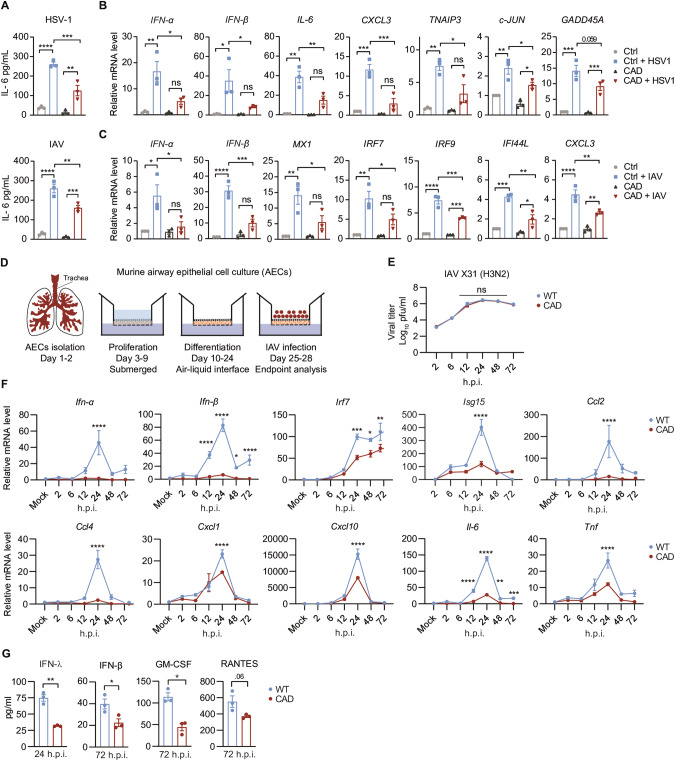
CAD-dependent pro-inflammatory responses in epithelial cells during viral infection. **A**–**C** CAD-regulated response of HeLa cells to viral infection. **A** Secretion of IL-6. HeLa-supernatants cells were analyzed by ELISA after 16 h of infection with HSV-1 (MOI = 1) or after 24 h of infection with IAV (MOI = 10). HeLa control cells (non-targeting control gRNA) and HeLa CAD-deficient cells (CAD deletion by CRISPR/Cas9) were infected in parallel. **B**, **C** HeLa cells were infected with HSV-1 (**B**) or IAV (**C**) as in (**A**), and the expression of virus-response genes was determined by qPCR. Data are means/SEM from three independent experiments (two technical replicates each). Fold change of RNA-expression was calculated after normalizing the values of treatment group to uninfected controls. Statistics was performed using one-way ANOVA with Tukey’s test for multiple comparison correction. **D**–**G** Mouse airway epithelial cells (AEC) were isolated from wt or CAD-deficient mice. **D** Experimental setup. **E** Viral titers were determined in culture supernatants at the time points of infection indicated. **F** Relative mRNA expression of interferon stimulated genes and cytokines during viral infection. Fold change was calculated after normalizing the values of treatment group with uninfected controls. Statistical analysis was performed using two-way ANOVA, corrected for multiple comparisons by using the Sidak method. **G** Cytokine levels in basal media were measured by ELISA. Statistical analysis was done by unpaired *t*-test. Data are means of three transwell replicates. Error bars represent SEM. For all samples: *p* < 0,05: *, *p* < 0,01: **, *p* < 0,001: ***, *p* < 0,0001: ****.

We used a DNA virus (Herpes simplex virus 1, HSV-1) and an RNA virus (influenza A virus, IAV) to test for the contribution of CAD to the response to infection. We first infected control and CAD-deficient HeLa cells with these viruses and measured the response. Infection with either virus induced the secretion of IL-6, and IL-6 levels were reduced when CAD-deficient cells were infected (Fig. 5A). HSV-1 can activate CAD through sub-lethal signals but can in addition cause a BAX/BAK-independent DDR [9]. This DDR is likely to involve ATM/ATR, which in turn may cause a caspase-independent activation of CAD [16]. Whichever the mechanism of CAD-activation, genes known to be induced by HSV-1-infection were also less strongly induced in CAD-deficient cells (Fig. 5B). The expression of IAV-induced genes such as the interferon-responsive genes *MX1* and *IFI44L* and other response genes was also reduced in IAV-infected CAD-deficient cells compared to control cells (Fig. 5C; it is worth pointing out that IAV in these conditions induces very little apoptosis (1–2%) [9]).

We next infected primary mouse airway epithelial cells (AECs), isolated from wt and CAD-deficient mice, with IAV. AECs are differentiated in vitro at an air-liquid interface and can serve as a model for infection with respiratory viruses [33] (Fig. 5D). We monitored IAV-infection by measuring expression of a viral gene and found no difference between wt and CAD-deficient cells (Fig. 5E). However, the induction of immune response genes was strongly diminished in cells lacking CAD. We observed a reduced induction of *Ifn-α* and *-β* genes as well as IFN-responsive genes. Transcripts for *Il-6* and *Tnf* and several chemokines were also reduced in cells lacking CAD (Fig. 5F). The levels of IFN-λ, IFN-*β*, GM-CSF and RANTES were lower in supernatants of infected CAD-deficient cells compared to infected wt AECs (Fig. 5G). The results show that CAD contributes to pro-inflammatory gene induction during viral infections.

### CAD-deficient mice have an impaired defense-reaction against IAV-infection

**Fig. 6:**
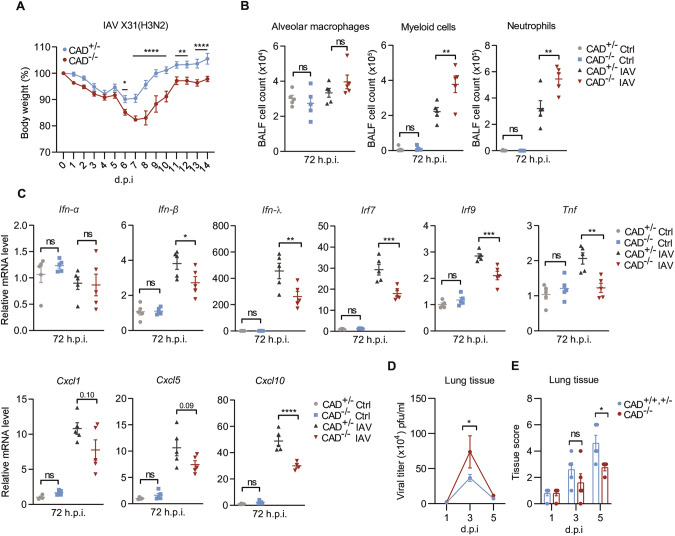
CAD contributes to inflammation and immune defense during Influenza A virus infection in mice. **A** Changes in body weight in CAD-deficient mice and heterozygous littermates during IAV infection (means/SEM of six mice per group). Statistical analysis was performed using two-way ANOVA, corrected for multiple comparisons by using the Sidak method. **B** Immune cell-infiltration during IAV-infection as measured in bronchoalveolar lavage fluid (BALF) at 72 hours of IAV infection. BALF cells were analyzed by flow cytometry to identify the populations indicated. Each mouse is shown as one symbol. **C** Expression of interferon stimulating genes (ISGs) and other pro-inflammatory mediators in murine lung tissues during IAV infection. For B and C, data are means of values from five individual mice per group. Statistical testing was performed using one-way ANOVA, corrected for multiple comparisons by Tukey test. Fold change was calculated after normalizing the values of treatment group with uninfected control group. **D,** **E** Viral titers and histopathological score of lung tissues at different time points following viral infection. Mixed-effects model analysis was performed rather than two-way ANOVA due to a missing value in the CAD^-/-^ group at day 5. Data are means of values from five mice. Error bars represent SEM. For all samples: *p* < 0.05: *, *p* < 0.01: **, *p* < 0.001: ***, *p* < 0.0001: ****.

CAD-deficient mice have no obvious abnormality [13]. We infected CAD-deficient mice and heterozygous littermates with IAV through the respiratory tract. Weight loss was more pronounced in infected CAD-deficient mice than in heterozygous mice, and recovery was delayed (Fig. 6A). At 72 h post-infection, there was no change in alveolar macrophage numbers but we found increased numbers of neutrophils in the brochoalveolar lavage fluids in CAD-deficient animals (Fig. 6B). In lung tissue from these mice, mRNA-levels of *Ifn-*λ*, Ifn-β, Tnf* and other inflammatory factors were significantly reduced (Fig. 6C). Little virus was recovered from the lungs in either mouse strain on days 1 and 5. On day 3 post-infection, relatively high titers of IAV were found, and the amount of virus was significantly higher in lung tissue from CAD-deficient mice compared to heterozygous mice (Fig. 6D). When we examined the lung tissues of infected mice on day 5, we found significantly less tissue damage in lungs from CAD-deficient animals (Fig. 6E, Fig. S8); this latter effect is consistent with the well-documented inhibition of lung damage-repair in IAV-infection by interferons [34]. The results of this study show that non-lethal CAD-activation, which occurs through signals of the mitochondrial apoptosis pathway, leads to a pro-inflammatory response that drives host-defense reactions to microbial infection.

## Discussion

Caspase-dependent CAD-activation by infection has been reported earlier. We here suggest a physiological role of this activation in the induction of pro-inflammatory genes and a contribution to host defense through the DDR. cGAS-STING-IFN and other major signaling pathways are activated downstream of CAD, causing the pronounced induction of pro-inflammatory genes. In the absence of CAD, the inflammatory response to viruses, and the defense against IAV, is impaired. The results link sub-lethal mitochondrial signals, DNA-damage and genomic stress to the immune response and suggest that ‘intentional’, self-inflicted damage to the genomic DNA is an integral feature of host defense.

The inflammatory nature of the DDR is well known, and numerous studies have shown that chemically or physically DNA-damaging agents cause a pro-inflammatory DDR. The features of sunburn are at least partly the results of the DDR to UV-induced DNA-damage [35]. Earlier studies have focused on DDR-kinases, in particular ATM [21, 30], but a role of STING in the immune recognition of DNA-damage has also been reported [30]. It may be hypothesized that the loss of ATM impairs DNA-repair, explaining the higher level of STING-activity in ATM-deficient mice [30]. DNA-damage itself appears to be induced by CAD during infection but because CAD can be spontaneously active [17], CAD-activity may also induce a low-level DDR at steady state.

CAD led to the activation of cGAS and also induced a cGAS-STING-independent response, indicating a complex network of response signals. Inhibition of individual kinases had relatively minor effects on cytokine/chemokine secretion but a contribution of ATM, ATR, p38 and RIPK1, as well as of NF-κB, was detectable. The contribution of kinase signaling pathways to physical and chemical DNA-damage seems variable: etoposide-induced TNF-production required ATM and RIPK1 [21] while UV-light caused massive p38-dependent gene-induction [19]. The activation of cGAS by CAD appears to have broad implications: cGAS may directly recognize viral DNA but cGAS-activation has also been shown during RNA-viral infection [36, 37]. While this may receive a contribution from mtDNA [36], CAD is activated by sub-lethal signals from DNA- and RNA-viruses as well as by bacteria [9, 10]. CAD-dependent STING-activation may therefore expand the role of cGAS/STING in the recognition of infection considerably. The precise nature of the cGAS-ligand generated by CAD-activity is unclear. CAD-activity can generate micronuclei [8, 17], which can serve as ligands for cGAS [38]. Cytosolic chromatin fragments (CCF), which can also be generated as a consequence of DNA-damage and act as cGAS-ligands [38] are a further possibility. Loss of ATM increases the cytosolic presence of nuclear DNA [30], suggesting that ‘spontaneous’ DNA-damage, which may include spontaneous CAD-activity [17], leads to some form of DNA-translocation into the cytosol. The precise mechanism of this event however will have to be worked out.

The data further suggest that DNA-damage is a desirable event during infection. DNA-damage is potentially dangerous: in the case of faulty repair, mutations arise that may compromise gene function and may lead to cancer. On the other hand, DNA-damage repair is hardly ever faulty. DNA-damage is a very common event in human cells, in response both to exogenous (such as radiation) and to endogenous stimuli (for instance replication stress) [39, 40]. Because mammalian cells are equipped with tremendously powerful machineries that detect and repair DNA-damage [41], most DNA-damaging events remain inconsequential in terms of mutation. Even if all infections regularly activate CAD, as seems to be the case, the resulting DNA-damage will in most cases be repaired without trace. Often repeated, or chronic, infections may eventually cause persistent mutations. It is well established that cells in human tissues accumulate mutations throughout life [42], and a contributing factor to this genomic damage may be the activity of CAD. Chronic infections are associated with cancer [43]. While some infectious agents are very prominent as carcinogenic agents, such as *Helicobacter pylori* and human papilloma virus, epidemiological studies have associated many other infectious agents with increased risk of cancer, for instance *Salmonella* Typhi [44], *Mycobacterium tuberculosis* [45] and *Chlamydia trachomatis* [46]. This risk, which can be identified at the population level, may be a reflection of repeated CAD-activation by infectious agents.

During infection, CAD is activated in a caspase-dependent fashion in the mitochondrial apoptosis pathway [9, 10]. Mitochondria have a number of functions in mammalian cells. One of the functions of mitochondrial apoptosis is in danger-recognition, when a potentially dangerous stimulus is received by the cell, and the cell activates the apoptosis apparatus to die in response [47]. A pro-inflammatory function of mitochondria has become a focus of research in recent years [48]. Our results add a new type of mitochondrial pro-inflammatory activity, through sub-lethal activation of caspases and CAD. It should be noted that this also attributes a pro-inflammatory function to cell death caspases. During apoptosis, these enzymes are required to silence inflammation [49]. During sub-lethal signaling processes, they however have a pro-inflammatory function through the activation of CAD. Sub-lethal signals are likely to occur at least as widely as lethal, apoptotic signals. The pro-inflammatory function of sub-lethal signals therefore may be important in many situations. Therapeutic interference with this pathway may have beneficial effects in inflammatory diseases.

## Materials and methods

### Cell lines and cell culture

HeLa229 cells (ATCC CCL-2.1) were cultured in RPMI 1640 medium (61870044, Thermo Fisher Scientific) with 10% FCS (F7524, Sigma or AC-SM-0190, Anprotec), 1% penicillin/streptomycin (15140130, Thermo Fisher Scientific). HaCaT keratinocytes [50] were cultured in DMEM (41965062, Thermo Fisher Scientific) supplemented as above. Cell lines were tested negative for mycoplasma contamination using two different kits: Venor®GeM or Lonza MycoAlert™ mycoplasma detection kit. Gene-deficient cells were generated via CRISPR/Cas9 genome editing by transducing the cells with the lentiviral vector lentiCRISPR v2 (Addgene) and selection with puromycin (ant-pr-1, Invivogen). gRNA used for CAD, BAX, BAK or control have been described earlier [9]. gRNAs for STING (GCTGGGACTGCTGTTAAACG), cGAS (ATGATATCTCCACGGCGGCG), ATM (CCAAGGCTATTCAGTGTGCG) and ATR (GATGCTTTGATTTATATGCA) were taken from the Brunello gRNA database [51]. Generation of HeLa ICAD-mAID-GFP cells was done as described for HaCaT cells [17]. HaCaT ICAD-mAID-GFP cells expressing a NF-κB-Luciferase system were obtained by stable transduction of the cells using a lentivirus [52].

### Reagents and antibodies

Auxin (IAA, 3-indoleacetic acid, Sigma, I2886) was dissolved in DMSO and used as indicated. HaCaT cells were treated with 20 µM auxin (HeLa cells: 5 µM). Inhibitors (all dissolved in DMSO) were used at the following concentrations: RIPK1 (Necrostatin-1, N9037, Sigma): 10 µM; ATM (KU55933, S1092, Selleckchem): 10 µM; p38 (SB203580, S1076, Selleckchem): 10 µM; IKK1/2 (BMS-345541, B9935, Sigma): 5 µM. The inhibitors were added one hour (Necrostatin-1 30 min) before stimulation with auxin and were added again after the washout of auxin after 6 hours as indicated. ATM inhibitor was added after 6 h of auxin stimulation. ABT-737 (10 µM, S1002) and S63845 (50-100 nM, S8383) were both from Selleckchem. Propidium iodide (P4170, Sigma), DAPI (D9542, Sigma). Hydrogen peroxide (H_2_O_2_, Sigma, H1009), N-acetyl-L-cysteine (NAC, Bio-Techne, #7874). Primary antibodies used were: from Cell Signaling: anti-ATM (#2873), anti-ATR (#13934), anti-BAK (#12105), anti-BAX (#2772), anti-γH2A.X (WB, #2577; for immunostaining #9718), anti-ICAD (#9732), anti-cGAS (#79978), anti-STING (#13647), anti-phospho-STING (#19781), anti-phospho-SAPK/JNK (#4668), anti-phopho-p38 MAPK (#4511), anti-phospho-p44/42 MAPK (ERK1/2, #4370), anti-p53 (#2524), anti-phospho-p53 (#9284), anti-phospho-IRF3 (#37829), anti-phospho-p65 (#3033), anti-NF-κB p100/p52 (#3017). Anti-phospho-ATM (ab81292, Abcam), anti-phospho-IRF3 (ab76493, Abcam), anti-phospho-ATR (GTX128145, GeneTex), anti-GAPDH (MAB374, Millipore), 53BP1 (ab36823, Abcam), RAD51 (NB100-148, Novus) and anti-MX1 (Clone M143, provided by G. Kochs, Freiburg). Secondary antibodies were anti-Rabbit IgG (H + L) HRP (A6667, Sigma-Aldrich), anti-mouse IgG (H + L) HRP (115-035-166, Dianova), Alexa Fluor 647 (111-605-144, Jackson ImmunoResearch Laboratories Inc.) or Cy5 (715-175-151, Dianova).

### Western blotting

Cells (1.5–2.5 × 10^5^) were seeded in 6-well plates. Cells were washed in DPBS (14190169, Thermo Fisher Scientific) and lysed with 1X Laemmli buffer or RIPA buffer in the wells. Lysis with RIPA buffer was followed by protein measurement using DC Protein Assay (5000112, Bio-Rad). Samples were run on SDS-PAGE gels (4-20% gradient NOVEX gels, XP04205). PVDF (10600021, GE Healthcare) membranes were blocked with 5% milk in TBST. Proteins were detected with ECL substrate. Signal intensity was calculated with Image J as described in the figure legends using GAPDH for normalization.

### Cell death assay

Per well, 1.5–2.5 × 10^5^ cells in 6-well plates were stimulated with auxin for 24 h. Cells were trypsinized and washed with PBS and cells were immediately stained with propidium iodide (PI) and analyzed by flow cytometry (FACS Calibur, BD). For AnnexinV/7-AAD staining, HeLa cells were treated with ABT-737 10 μM and S63845 50-100 nM for 72 h. Cells were collected as described above and stained with Annexin V-FITC (Invitrogen BMS306FI-300) and 7-AAD (559925, BD Pharmingen) in 1x Annexin V-binding buffer (556454, BD Pharmingen) and analyzed in LSRFortessa Cell Analyzer (BD Biosciences). For quantification of absolute cell numbers, 123count eBeads counting beads (ThermoFisher Scientific) were used.

### Cell cycle analysis

HeLa ICAD-mAID-GFP cells were seeded in a 6-well plate at a density of 2.5 × 10^5^ per well (6 h and 6 h + 24 h time points) or 1.5 × 10^5^ per well (6 h + 48 h). The next day, the cells were treated with auxin (5 μM) for 6 h. For some samples, auxin was washed out after 6 h and culture was continued for 24 h or 48 h. Cells were washed once with PBS and trypsinized. Equal numbers of cells (4 × 10^5^) were pelleted down in a 96-well conical plate and fixed for 1 h in 4% formaldehyde in PBS at 4 °C. Fixed cells were washed twice with PBS and cells were resuspended in 200 µL Permeabilization Buffer (00-5523-00 Thermo Fisher) and incubated for 1 h at 4 °C. Staining was done in Permeabilization Buffer using anti-γH2A.X antibody (γH2A.X-Alexa Fluor 647 1:200 (#9720, Cell Signaling)) over night at 4 °C. Cells were again washed twice with Permeabilization Buffer and resuspended in PBS containing 1:2000 DAPI (D9542, Sigma) and incubated for 15 min at room temperature. Samples were analyzed by flow cytometry using a LSRFortessa Cell Analyzer (BD Biosciences). The acquired data were analyzed with FlowJo software v10.7.1.

### Immunofluorescence microscopy

Cells were seeded onto a treated 8-well ibidi chamber (3 × 10^4^ cells). The next day, cells were treated with auxin for 6 hours. Cells were fixed with PFA (4% in PBS) for 10-15 minutes at room temperature, followed by permeabilization and blocking in PBS/5% (w/v) BSA/0.5% (v/v) Triton X-100) for 45 minutes at room temperature. Incubation with γH2AX-antibody (9718, Cell Signaling, 1:400) was performed overnight at 4 °C in PBS/1% (w/v) BSA/0.1% (v/v) Triton X-100. Wells were washed three times in wash buffer (PBS/0.1% (v/v) Tween-20). Incubation with secondary antibody (1:500) conjugated with Alexa Fluor 647 (111-605-144, Jackson ImmunoResearch Laboratories Inc.) was performed in the dark for two hours at room temperature in the presence of DAPI (1:10.000). Wells were washed three times with wash buffer and ibidi mounting media was added to each well. Cells were imaged with a Zeiss LSM 710 (magnification: 63x in oil). For 53BP1 and RAD51 immunostaining, cells were seeded onto a treated 8-well ibidi chamber (2 × 10^4^ cells). The next day, cells were treated with auxin for 6 hours. After incubation, cells were either fixed (6 h time point) or washed with warm media and incubated for additional 24 hours in complete media. Cells were fixed with PFA (4% in PBS) for 10 min at room temperature, followed by permeabilization and blocking in PBS/5% (w/v) BSA/0.5% (v/v) Triton X-100) for 1 hour at room temperature. Incubation with 53BP1 (ab36823, Abcam, 1:400) and RAD51 (NB100-148, Novus, 1:200) was performed overnight at 4 °C in PBS/1% (w/v) BSA. Wells were washed three times in wash buffer (PBS/0.2% (v/v) Tween-20). Incubation with secondary antibody (1:500) conjugated with Alexa Fluor 647 (111-605-144, Jackson ImmunoResearch Laboratories Inc.) or Cy5 (715-175-151, Dianova) was performed in the dark for one hour at room temperature in the presence of DAPI (1:10.000). Wells were washed three times with wash buffer and ibidi mounting media was added to each well. Cells were imaged with a Zeiss LSM 710 (magnification: 63x in oil).

*ELISA* Cytokines in supernatants of HaCaT, HeLa and murine airway epithelial cells were measured using the following kits: human IL-6 (430516, BioLegend), human IL-8 (431504, BioLegend), human CXCL-1 (DY275-05, R&D Systems), LEGENDplex Human Type 1/2/3 Interferon Panel (5-plex) (740396), LEGENDplex Mouse Anti-Virus Response Panel (13-plex) (740622, Biolegend), murine IL-28A/B (IFN-λ2/3) (DY1789B-05 R&D Systems). The 2′, 3′-cGAMP levels in total cell lysates of HaCaT ICAD-mAID-GFP and HeLa ICAD-mAID-GFP cells were measured using the DetectX 2′, 3′-Cyclic GAMP Enzyme Immunoassay Kit (Arbor assays, K067-H1) according to the manufacturer’s instructions. Briefly, 2 × 10^6^ cells were seeded and treated with Auxin as described in the figure legends. Cells were trypsinized, counted, and lysed in Lysis Buffer (20 mM HEPES (pH 7.8), 100 mM NaCl, 5% glycerol, 0.5% NP-40, 5 mM EDTA, 1 mM dithiothreitol and phosphatase and protease inhibitors) and further lysed by sonication as described previously [53].

### NF-κB reporter assay

After stimulation, cells were harvested, washed with DPBS and counted, and 4 × 10^3^ cells were measured in a 96-well plate using the ONE-Glo™ Luciferase Assay System (E6110, Promega) and a TECAN reader. Luminescence was normalized to the untreated control.

### Primary peripheral blood neutrophils

Human neutrophils were obtained from peripheral blood of healthy adult volunteers by negative selection with a magnetic cell separation system (EasySep Direct human neutrophil isolation kit, Stem Cell Technologies). Purity of cell preparations was confirmed by Giemsa staining.

### Transwell migration assay

A 24-well transwell system (3 µm pore, Corning Costar) was used. HeLa cell supernatants (400 µl) were added as indicated into the lower chamber, and 2 × 10^5^ freshly isolated neutrophils in 200 µl complete medium were placed into the upper chamber. Negative controls (medium) and positive controls (human IL-8 (5 ng/ml)) were included. After 45 min incubation, migration was stopped. Migrated cells in the lower chamber were harvested and quantified using an automatic cell counting system (CASY cell counter, Omni Life Science).

### Neutrophil survival

Neutrophils (3 × 10^5^/300 µl) were co-incubated for 22 hours with supernatants from HeLa cells treated with auxin as indicated. Cells were harvested, stained with Annexin V-FITC (BMS306FI, Invitrogen) and propidium iodide (PI) and analyzed by flow cytometry (FACS Calibur, BD).

### Alkaline and neutral comet assay

HaCaT cells were seeded in a 6-well plate at a density of 2.5 × 10^5^ per well. The next day, the cells were treated with auxin (20 μM) for 6 h. For some samples, auxin was washed out after 6 h and culture was continued for 24 h or 48 h. Cells were washed once with DPBS and trypsinized. Cells were counted in a CASY cell counter (OMNI Life Science), centrifuged at 480 xg for 5 minutes, the pellet was washed once with PBS and centrifuged again. The pellet was resuspended in PBS to obtain a cell concentration of 5 × 10^5^/ml. Cells were then subjected to either Alkaline or Neutral Comet Assay (4250-050-K, R&D Systems) according to the manufacturer’s instructions with the following modifications. The cell suspension for agarose bedding was used at a concentration of 5 × 10^5^ cells/ml instead of 1 × 10^5^ cells/ml. For the Alkaline Comet Assay, lysis and unwinding incubations were increased to 60 minutes. Electrophoresis was performed in an electrophoresis chamber (CSL-COM20, Cleaver Scientific Ltd) with the alternative electrophoresis buffer, as described in the manufacturer’s protocol. For temperature control, the electrophoresis setup was connected to a cooling aggregate and electrophoresis was performed for 30 minutes at a constant 25 V and 300 mA. For the Neutral Comet Assay electrophoresis was performed using the manufacturer’s instructions for alternative electrophoresis units. Slides were dried for up to 60 minutes. DAPI was used for the staining, at a concentration of 1:1000 and incubated for 15 minutes. Imaging of the slides was done using a Keyence microscope (BZ-9000, Keyence). For each biological independent experiment (*n* = 3), 6-9 pictures were taken per sample, covering the whole sample area of the slide. Pictures were then analyzed with Image J using the Open Comet Plugin.

### RNA isolation, RNA-seq. and analysis

Library preparation for bulk 3′-sequencing of poly(A)-RNA was done as described [54]. Barcoded cDNA was generated using a Maxima RT polymerase (Thermo Fisher) with oligo-dT primer containing barcodes, unique molecular identifiers (UMIs) and an adapter. 5′ ends of the cDNAs were extended by a template switch oligo (TSO) and after pooling of all samples full-length cDNA was amplified with primers binding to the TSO-site and the adapter. NEB Ultra II FS kit was used for fragmentation of cDNA. After end repair and A-tailing a TruSeq adapter was ligated and 3′-end-fragments were finally amplified using primers with Illumina P5 and P7 overhangs. The published protocol [54] was modified to exchange P5 and P7 sites to allow sequencing of the cDNA in read1 and barcodes and UMIs in read2 for better cluster recognition. The library was sequenced on a NextSeq 500 (Illumina) with 75 cycles for the cDNA in read1 and 16 cycles for the barcodes and UMIs in read2. Data were processed with the published Drop-seq pipeline (v1.0) to generate sample- and gene-wise UMI tables [55]. Reference genome (GRCh38) was used for alignment. Regularized log transformation [56] of library size normalized gene expression [57] were used to depict different treatments and time points using principal component analysis (PCA) plot. For RNA-expression analysis from cells and lung tissues, samples were lysed in TRI Reagent and RNA was extracted using the Direct-zol RNA MicroPrep or RNA Miniprep kit (R2061/R2053, Zymo Research) according to the manufacturer’s instructions. Lung tissues were harvested and homogenized using Fastprep-12 homogenizer (MP Biomedicals) before RNA extraction. RNA concentrations were normalized before cDNA synthesis. RNA was reverse transcribed using RevertAid First Strand cDNA synthesis kit (K1622, Thermo Fisher Scientific) or Transcriptor first strand cDNA synthesis kit (04379012001, Roche) and analyzed by QuantStudio 5 Real-Time PCR detection system (Applied Biosystems) using 1X SYBR Green master mix (4472918, Applied Biosystems). Results were analyzed using QuantStudio Design and Analysis software v.1.5.2. The list of primers used for genes of interest are given in the table. The comparative C_T_ method (ΔΔCT method) was used to determine relative expression of target gene to the house keeping gene [58].

### Protein-protein interaction (PPI) network and pathway enrichment analysis

First, 647 significantly upregulated genes were identified out of total 15,295 expressed genes through transcriptome analysis after 14 hours of CAD activation (Cutoff Log_2_ fold change >0.6, P adj. value < 0.05). Then, functional interactions of these filtered genes were analyzed to extract underlying signaling pathways in the context of protein-protein interactions (PPIs). For that, STRING database (https://string-db.org) was used to generate a network of 936 interactions between 179 proteins, containing 37 additional interactomes. These functional interactions were extracted using STRING-resources (databases, networks, experiments, textmining, neighborhood, fusion, co-occurence and co-expression of interactomes). For functional and topological analysis of a PPI-network, cytoscape v3.8.2 software was used to identify hub proteins/core interactomes with higher degrees of interaction, fold increase and betweeness centrality within the network. Pathway enrichment analysis of these interactomes was performed using Metascape database (http://metascape.org) as previously described [32]. P value was adjusted to <0.05 to consider only statistically significant pathways. The information of significantly upregulated interferon-regulated genes (IRGs) in our gene expression datasets was extracted using all available resources (in vitro/in vivo data of *Homo sapiens*) on INTERFEROME v2.0 database [59]. While, innate immune response genes were identified through InnateDB curated genes database (https://www.innatedb.com) [60].

### HeLa cell culture and infection

CAD-deficient HeLa cells and non-targeting gRNA control HeLa cells have been previously described [9]. Influenza A virus (IAV) (A/WSN/1933 - H1N1) and HSV-1 (strain McIntyre - ATCC) were kindly provided by Georg Kochs, Institute of Virology, Freiburg. For IAV-infection, 5 ×10^5^ cells per well were seeded in 6-well plates. After 24 h, cells were infected for 2 hours with IAV (MOI = 10) or HSV-1 (MOI = 1) in 1 ml DMEM medium containing 1% FCS. Then, the medium was replaced by fresh DMEM medium with 10% FCS. IAV and HSV-1 infected cells were incubated for 24 hours and 16 hours, respectively. Cells and supernatants were harvested for further analysis.

### Primary murine airway epithelial cells (AECs) culture and infection

Primary mouse airway epithelial cells culture was performed as previously described [33]. Tracheas were harvested from wt and CAD-deficient mice (C57BL/6). After enzymatic digestion of the tissue and separation of fibroblasts, cells were seeded onto collagen-coated transwells with 0.4 µm pore size in a 24 well plate. Once the cells were confluent, the liquid from the upper chamber was removed to initiate air-liquid interface (ALI) for epithelial cell differentiation. The cells were infected with IAV (strain A/X-31 H3N2) MOI = 1 after two weeks of ALI-culture. Cell lysate, basal media samples and PBS-wash/supernatant were taken at various time points for the measurement of gene expression, cytokine profiling or viral titer.

### Mouse models of viral infection

The animal studies were approved by the authorities in charge (Regierungspräsidium Freiburg). In vivo experiments were conducted with 8-22 weeks old CAD-deficient mice and heterozygous littermates on a C57BL/6 background. CAD-deficient mice were kindly provided by Prof. Roberto Caricchio, Philadelphia with the permission of Prof. Shigekazu Nagata, Osaka. All mice were bred and maintained under specific pathogen free conditions at the animal facility of preclinical research at the University hospital of Freiburg. Animal handling and treatment was conducted according to the guidelines of the Federation of European Laboratory Animal Science Associations (FELASA) and the national animal welfare authority (GV-SOLAS). All in vivo experiments were performed in accordance with the German animal protection law (TierSchG) and authorized by animal welfare committee of the University of Freiburg and local government’s animal welfare committee (Regierungspräsidium Freiburg. G-19/057). Mouse genotypes were confirmed via the KAPA HotStart mouse genotyping kit (KAPA biosystems, Boston, MA, USA) and gene-specific primers. The mice were anesthetized using ketamine/xylazine and infected intranasally with IAV (strain A/X-31 H3N2) 5 ×10^2^ pfu in a volume of 40 µl PBS. For endpoint analysis, mice were sacrificed by cervical dislocation. Bronchoalveolar lavage (BAL) fluid was taken for immune cell characterization and cytokine profiling by flushing lungs 3 times with 800 µl PBS. To determine the viral titer in lungs, the left lobe of lung tissue was collected in 800 µl PBS and homogenized using a FastPrep-24 homogenizer (MP Biomedicals). After homogenization, the samples were centrifuged for 10 min. at 6000 xg and 4 °C and supernatant was collected to determine viral titer by viral plaque assay. The inferior lobe of the right lung was collected, fixed with 4% formaldehyde at 4 °C for 24 hours and stored in 70% ethanol for further processing to examine the histopathology. The degree of histopathological changes was assessed by applying a semi quantitative scoring system [61] on lung sections (2 µm thick) of formalin-fixed, paraffin-embedded (FFPE) tissue. From the right lung, superior, middle and post caval lobes were taken for RNA isolation and RT-qPCR. For LD50 calculation, the mice were infected in four groups with IAV (10^2^, 10^3^, 10^4^ and 10^5^ pfu) as above and monitored for 14 days. Infected mice were sacrificed if their weight fell below 75% of their initial body weight. The LD50 was calculated using the Reed-Muench method [62].

### Flow cytometry analysis of BAL fluid

Inflammatory cell populations from bronchoalveolar lavage (BAL) fluid of murine lungs were isolated and characterized according to a previously described protocol [63]. The following antibodies and regents were used: rat anti-mouse CD16/CD32 Fc Block (clone 2.4G2) (BD Pharmingen); Fixable Viability Dye eFluor 780 (eBioscience); APC-R700 siglec (clone E50-2440) (BD Horizon); biotin anti-mouse Ly-6G antibody (clone 1A8) coupled with Brilliant Violet 421 streptavidin (BioLegend); Brilliant Violet 711 anti-mouse/human CD11b antibody (clone M1/70) (BioLegend); PE hamster anti-mouse CD11c (clone HL3) (BD Pharmingen); V500 rat anti-mouse I-A/I-E MHCII (M5/114) (BD Horizon); FITC hamster anti-mouse CD3e (clone 145-2C11) (BD Pharmingen). The samples were fixed with 2% PFA and analyzed in LSRFortessa Cell Analyzer (BD Biosciences). For quantification of absolute cell numbers, 123count eBeads counting beads (ThermoFisher Scientific) were used. The acquired data were analyzed with FlowJo software v10.7.1. We identified myeloid cells as CD11c^-^/CD11b^+^/Ly6G^-^; neutrophils as CD11b^+^/Ly6G^+^; alveolar macrophages as CD11c^+^/SiglecF^+^/MHC II^+^.

### Statistical analysis

Statistical analysis was performed using GraphPad Prism v8.0.2. Statistical tests used are indicated in the figure legends. Unpaired *t*-test or Mann–Whitney test was used when comparing two groups. One-way and two-way ANOVA and Sidak’s or Tukey’s post-hoc tests were used for multiple testing.

### Primers used for this study

***Murine***

**Table:** 

Targets	Primers
*Ccl2*	forward 5′-CCTGCTGCTACTCATTCACCA-3′ reverse 5′-ATTCCTTCTTGGGGTCAGCA-3′
*Ccl4*	forward 5′-TTCCTGCTGTTTCTCTTACACCT-3′ reverse 5′-CTGTCTGCCTCTTTTGGTCAG-3′
*Cxcl1*	forward 5′-CTGGGATTCACCTCAAGAACATC-3′ reverse 5′-CAGGGTCAAGGCAACCCTC-3′
*Cxcl5*	forward 5′-GTTCCATCTCGCCATTCATGC-3′ reverse 5′-GCGGCTATGACTGAGGAAGG-3′
*Cxcl10*	forward 5′-AAGTGCTGCCGTCATTTTCT-3′ reverse 5′-GTGGCAATGATCTCAACACG-3′
*Gapdh*	forward 5′-AGGTCGGTGTGAACGGATTTG-3′ reverse 5′-TGTAGACCATGTAGTTGAGGTCA-3′
*Ifn-α*	forward 5′-CCTGAGAAGAGAAGAAACACAGCC-3′ reverse 5′-GGCTCTCCAGACTTTCTGCTCTG-3′
*Ifn-β*	forward 5′-GCTCCTGGAGCAGCTGAATG-3′ reverse 5′-CGTCATCTCCATAGGGATCTTGA-3′
*Ifn-λ*	forward 5′- AGCTGCAGGCCTTCAAAAAG-3′ reverse 5′- TGGGAGTGAATGTGGCTCAG-3′
*Il-6*	forward 5′-TAGTCCTTCCTACCCCAATTTCC-3′ reverse 5′-TTGGTCCTTAGCCACTCCTTC-3′
*Irf7*	forward 5′-GAGACTGGCTATTGGGGGAG’3 reverse 5′-GACCGAAATGCTTCCAGGG-3′
*Irf9*	forward 5′-GCCGAGTGGTGGGTAAGAC’3 reverse 5′-GCAAAGGCGCTGAACAAAGAG-3′
*Isg15*	forward 5′-GAGCTAGAGCCTGCAGCAAT-3′ reverse 5′-TTCTGGGCAATCTGCTTCTT-3′
*Tnf*	forward 5′-CAGGCGGTGCCTATGTCTC-3′ reverse 5′-CGATCACCCCGAAGTTCAGTAG-3′

***Human***

**Table:** 

Targets	Primers
*c-JUN*	forward 5′-GAGCTGGAGCGCCTGATAAT-3′ reverse 5′-CCCTCC TGCTCATCTGTCAC-3′
*CXCL3*	forward 5′-GCAGGGAATTCACCTCAAGA-3′ reverse 5′-GGTGCTCCCCTTGTTCAGTA-3′
*GADD45A*	forward 5′-GAGAGCAGAAGACCGAAAGGA-3′ reverse 5′-CAGTGATCGTGCGCTGACT-3′
*GAPDH*	forward 5′-GAGTCAACGGATTTGGTCGT-3′ reverse 5′-GACAAGCTTCCCGTTCTCAG-3′
*IFI44L*	forward 5′-TCTGCCATTTATGTTGTGTGACA-3′ reverse 5′-CAGGTGTAATTGGTTTACGGGAA-3′
*IFN-α*	forward 5′-GACTCCATCTTGGCTGTGA-3′ reverse 5′-TGATTTCTGCTCTGACAACCT-3′
*IFN-β*	forward 5′-ATGACCAACAAGTGTCTCCTCC-3′ reverse 5′-GGAATCCAAGCAAGTTGTAGCTC-3′
*IL-6*	forward 5′-GGCACTGGCAGAAAACAACC-3′ reverse 5′-GCAAGTCTCCTCATTGAATCC-3′
*IRF7*	forward 5′-GCTGGACGTGACCATCATGTA-3′ reverse 5′-GGGCCGTATAGGAACGTGC-3′
*IRF9*	forward 5′-GCCCTACAAGGTGTATCAGTTG-3′ reverse 5′-TGCTGTCGCTTTGATGGTACT-3′
*MX1*	forward 5′-GGTGGTCCCCAGTAATGTGG-3′ reverse 5′-CGTCAAGATTCCGATGGTCCT-3′
*TNF*	forward 5′-CTCTTCTGCCTGCTGCACTTTG-3′ reverse 5′-ATGGGCTACAGGCTTGTCACTC-3′
*TNFAIP3*	forward 5′-TCCTCAGGCTTTGTATTTGAGC-3′ reverse 5′-TGTGTATCGGTGCATGGTTTTA-3′

### Supplementary information

Relevant supplementary file

Supplementary figures

Uncropped western blot figures

## Data Availability

RNA-seq. data have been deposited at the GEO database (accession number GSE212956, accessible using the following link (https://www.ncbi.nlm.nih.gov/geo/query/acc.cgi?acc=GSE212956) and token: ubknyieotjwvfih). All data are available in the main text or the supplementary materials.
