# Supplementary material for: The Caspase-Activated DNase drives inflammation and contributes to defense against viral infection
Source: Cell Death Differ. 2024 Jun 7;31(7):924–37. doi: 10.1038/s41418-024-01320-7 (PMC11239672; doi:10.1038/s41418-024-01320-7)
Supplement: Supplementary file 1 — Relevant supplementary file [file 41418_2024_1320_MOESM1_ESM.docx]

**Supplementary Data**

**Supplementary Fig. S1. Test for gene deficiency of the cells used in this study**

(**A**) Test for deficiency of CAD and cGAS in HeLa cells (referred to Fig. 1A, Fig. 5A-C and Suppl. Fig. S2). (**B**) Test for Bax/Bak, cGAS, and Sting deficiency in HaCaT and Hela cells used for experiments shown in Fig. 1D, and Fig. 3C-E, Fig. 4A-B. (**C**) Test for ATM, ATR and ATM/ATR deficiency in Hela ICAD-mAID-GFP cells used for experiments shown in Suppl. Fig. S6I.

**Supplementary Fig. S2. Contribution of non-lethal apoptotic stimuli towards pro-inflammatory cytokines secretion, cell death and DDR**

(**A**) HeLa cells (control or deficient in CAD) were treated with the BCL-2/BCL-XL/BCL-w-inhibitor ABT-737 (10 µM) or the Mcl-1 inhibitor S63845 (100 nM) for 72 h. Viable cell counts were determined using an automatic cell counter and analyzer system. Data are means/SEM of at least three experiments. Significance was determined by two-way ANOVA, Tukey’s post-hoc test. (**B, C**) HeLa cells (control or deficient in CAD) were treated with ABT-737 (10 µM) or the Mcl-1 inhibitor S63845 (50 nM) for 24 h, 48 h or 72 h. Cell viability was measured and analyzed through flow cytometry analysis. IL-6 concentrations were determined in the supernatants. Data are means/SEM of three experiments. Significance was determined by two-way ANOVA, Sidak’s post-hoc test. (**D-G**) HeLa cells (control or deficient in CAD) were treated with the Mcl-1 inhibitor S63845 (50nM or 100 nM) for 72 h. Non adherent cells/dead cells were removed (dead cells -), or not (dead cells +), through centrifugation before adding supernatants back to the cell culture at 48 h post-treatment. IL-6, IL-8 and CXCL-1 concentrations were determined in the supernatants after 24 h of dead cell removal. Viable cell counts were measured and analyzed through flow cytometry analysis. Data are means/SEM of four experiments. Significance was determined by two-way ANOVA, Sidak’s post-hoc test. (**H**) Indicated HeLa cells were treated with DMSO or S63485 (100 nM) for 65 hours. Cells were trypsinized, counted, lysed, and used for Western blot. (**I**) Graph shows band intensities normalized to GAPDH from three independent experiments represented as Means/SEM. Significance was determined by 2-way ANOVA, Sidak’s multiple comparison. (**J**) Western blots showing γH2AX expression after 6 h of ABT-737 (10 µM) treatment in HeLa cells (control or deficient in CAD). *p < 0.05; **p < 0.01; ***p < 0.001; ****p < 0.0001; ns, p ≥ 0.05.

**Supplementary Fig. S3. Experimental auxin system to activate CAD and the DDR.**

(**A**) ICAD-deficient HaCaT (HeLa) cells were transduced with a lentiviral construct expressing the auxin receptor TIR1 (Control) and human ICAD fused to mAID-GFP to obtain HaCaT ICAD-mAID-GFP cells. OsTIR1 (F-box protein) binds to endogenous Skp1 and forms the SCF^TIR1^(Skp1-Cullin1-TIR1) complex. In the presence of auxin, TIR1 binds to mAID^22^. SCF^TIR1^ ubiquitinates the mAID tag and promotes the degradation of mAID-tagged ICAD protein by the proteasome to activate CAD. (**B, C**) HaCaT (B) or HeLa cells (C) with a deletion in endogenous ICAD and expressing TIR1 (Control) were engineered to express an ICAD-mAID-GFP construct. Cells were treated with solvent (DMSO (-)) or with auxin (HaCaT: 20 µM, HeLa: 5 µM) over 24 h. Western blot shows loss of ICAD (fused to mAID-GFP) and the appearance of a DNA-damage response (γH2AX) over time. (**D, E**) The loss of ICAD as measured by flow cytometry as the loss of GFP-positive cells stimulated as in (B) and (C). (**F**) Western blots showing induction of DDR-signals after 6 h of auxin (5 µM) treatment in HeLa cells. Data shown are representative of two (B, C) experiments. (D, E) Shown are means/SEM of three independent experiments. (**G**) Cell cycle distribution of HeLa cells used in (C) by DAPI stain. Cells were treated for 6 h with solvent (DMSO (-)) or auxin (5 µM) and analyzed either directly or after 24 or 48 h following auxin wash-out. Cell cycle analysis was done by flow cytometry using DAPI-staining. (**H**) Cell cycle distribution of γH2AX positive and negative HeLa cells from the same experiment shown in (H) after 6h of auxin treatment. Cells were stained for γH2AX and cell cycle was analyzed either on γH2AX positive or γH2AX negative cells using DAPI. Data are shown in (G, H) as means/SEM of n=3 independent experiments. (**I**) HaCaT ICAD-mAID-GFP cells were treated with solvent (DMSO (-)) or auxin (20 µM). NAC (10 mM) was added 1 h before addition of auxin (6 h) or H_2_O_2_ (1 mM, 1 h). Cells were lysed and indicated proteins were analyzed by Western Blot. A Western Blot representative of three independent experiments is shown. (**J**) Neutral Comet Assay to assess DNA-damage. Cells were treated for 6 h with auxin (20 µM) and analyzed directly. Each symbol represents one cell. Data are from three separate experiments, and at least 70 nuclei were assessed per condition. Statistical analysis was done using nested t-test. Tail moment = tail DNA [%] x tail length.

**Supplementary Fig. S4.** **Non-lethal DDR in HaCaT and HeLa cells and cell death upon activation of CAD.**

(**A, B**) HaCaT or HeLa ICAD-mAID-GFP cells were seeded and stimulated the next day with DMSO or auxin (HaCaT: 20 µM; HeLa: 5 µM) for 6 h and stained for γH2A.X. The nucleus was detected by DAPI. Images were obtained with a Zeiss LSM 710 confocal microscope. Magnification 64x with oil. Scaling bars: 20 µm. (**C**) Quantification of γH2A.X-positive HeLa ICAD-mAID-GFP cells as shown in (B). In total, three separate experiments were analyzed. For each experiments, five random pictures were taken and a minimum of 70 cells were quantified per sample. On each picture, nucleus area was determined using DAPI, and the integrated density of the γH2AX-signal was measured using ImageJ. Graph shows the mean value/SEM of each experimental sample. Statistical analysis was done with a paired two-sample t-test. The threshold for positivity was defined as the mean values of the untreated samples plus three standard deviations. (**D**) HeLa ICAD-mAID-GFP cells were treated with solvent (DMSO) or auxin (5 µM) for 24 h. Cells were harvested and stained with propidium iodide (PI) for cell death and analyzed by flow cytometry. Shown are means/SEM of at least three experiments. Significance was tested by two-way ANOVA, Sidak’s post-hoc test. ***p < 0.001; ns, p ≥ 0.05.

**Supplementary Fig. S5. Transient DDR upon the activation of CAD.**

HaCaT (**A, C**) or HeLa (**B, D, E**) control cells and ICAD-mAID-GFP cells were treated with solvent (DMSO (-)) or auxin (HaCaT: 20 µM, HeLa: 5 µM) for 6 h. Auxin was washed out and culture was continued for 24 h (6 h+24 h) or 48 h (6 h+48 h). Cells were lysed and indicated proteins were analyzed by Western Blot. A representative Western Blot and quantification is shown as means/SEM of three independent experiments. * In (A) indicate cross-reactive bands of unclear origin. Significance was determined by two-way ANOVA, Tukey’s post-hoc test. Quantification: Signal intensity was calculated with Image J and normalized to the loading control. Values are normalized to the mean of all samples of each experiment. (**F-G**) HaCaT cells were treated with 20 µM auxin for 6 h and then washed with warm media. Fresh media was then added and the cells were further incubated for 24 h. Cells were fixed, 6 h after auxin treatment and 24 h after auxin removal. Cells were stained for 53BP1 and RAD51. Nuclei were stained with DAPI. A minimum of 50 cells per experiment (n=4 for 53BP1 and n=3 for RAD51) were analysed. Scale bar, 10µM. Significance was determined by two-way ANOVA, Sidak’s post-hoc test. *p < 0.05; **p < 0.01; ***p < 0.001; ****p < 0.0001; ns, p ≥ 0.05.

**Supplementary Fig. S6. CAD activates a pro-inflammatory DNA-damage response.**

(**A**) HeLa cells (control cells expressing only Tir1 or ICAD-mAID-GFP cells) were treated with solvent (DMSO (-)) or auxin (5 µM) for 6 h. Cells were lysed and proteins were analyzed by Western Blot (three separate experiments gave similar results). This experiment was conducted in parallel with experiments shown in Fig. S1F, and the membrane used for the four top panels was the same as the one probed for pATR for the data in Fig. S1F. (**B**) HaCaT ICAD-mAID-GFP cells were stably transduced with an NF-κB luciferase reporter lentivirus. Cells were treated with solvent or auxin for 6 h. Auxin was washed out and culture was continued for 24 h. As a positive control, cells were treated with TNF (100 ng). Luciferase activity was measured. Shown are means/SEM of four experiments (normalized to solvent-treated). Significance was tested by one-sample t-test. *p < 0.05; **p < 0.01. (**C**) HeLa cells (control or ICAD-mAID-GFP cells) were treated with solvent (DMSO (-)) or auxin (5 µM) for 6 h. Auxin was washed out and culture was continued for 48 h. Supernatants were collected and IL-6, IL-8 and CXCL-1 were measured by ELISA. Data are means/SEM of four experiments. Significance was determined by two-way ANOVA, Sidak’s post-hoc test. *p < 0.05; **p < 0.01; ***p < 0.001; ****p < 0.0001; ns, p ≥ 0.05. (**D**-**H**) HaCaT (D, F, H) or HeLa (E, G) cells (control or ICAD-mAID-GFP cells) were treated with auxin for 6 h, washed and culture was continued for 48h. Inhibitors of ATM, RIPK1, IKK or p38 were present during the entire time. IL-6, IL-8 and CXCL-1 were measured by ELISA in supernatants. Data are means/SEM of four experiments. Significance was tested by Welch’s t-test (E) or by two-way ANOVA, Sidak’s post-hoc test (F). *p < 0.05; **p < 0.01; ***p < 0.001; ****p < 0.0001. (**I**) HeLa ICAD-mAID-GFP cells (control, ATM, ATR, or ATM/ATR deficient cells) were treated with DMSO or auxin (5 µM) for 6 hours, then auxin was washed out and culture was continued for 24 hours. IL-6, IL-8 or CXCL-1 levels were measured in the supernatants by ELISA. Data are means/SEM of four experiments. Significance was tested by two-way ANOVA, Tukey’s post-hoc test. *p < 0.05; **p < 0.01; ***p < 0.001; ****p < 0.0001. n.d, values were below the detection limit.

**Supplementary Fig. S7. Gene expression and protein-protein-interaction networks in cells expressing active CAD.**

(**A**) Pathway enrichment analysis using the set of genes upregulated upon CAD-activation only in STING-deficient but not in control cells (see Fig. 4A, B). (**B**) Protein-protein interactions network (PPIN) of significantly upregulated gene data set (14 h auxin vs. solvent) indicating 936 interactions between 179 proteins containing 37 additional interactomes. (**C**) Functional interactions of filtered genes/proteins showing hub molecules with increased expression and higher interactions within protein-protein interactions network (PPIN). (**D**) Topology analysis showing core interactions with higher betweeness among proteins within the PPIN. (**E**) CAD drives a transcriptional IFN-response. Shown are the top 50 significantly upregulated type I interferon-regulated genes after 14 h of CAD activation in HaCaT ICAD-mAID-GFP cells. Heatmap diagram of log_2_ fold change values of gene expression at different time points with respect to their solvent controls after 6 and 14 h of CAD activation (see methods for details). Data are derived from RNA-sequencing experiments. DMSO, comparison of DMSO 14 vs. 6 h. (**F**) CAD regulates anti-viral defense IFN-signaling pathways. Pathway enrichment analysis was performed on expression of IFN-regulated genes from HaCaT ICAD-mAID-GFP cells stimulated with auxin or with solvent for 14 h. Significantly upregulated genes represented in protein-protein interaction networks were used to identify response pathways (see methods). P value <0.05 was adjusted to consider only statistically significant pathways.

**Supplementary Fig. S8.** **Histopathology of murine lung tissue following IAV infection.**

(**A**) H&E staining and (**B**) histopathological score of murine lung tissues at different time points following IAV infection. Data are representative of values from five individual mice per group except one missing value in CAD^-/-^ IAV-infected group at day 5. Statistical testing was performed using one-way ANOVA, corrected for multiple comparisons by Tukey test. Error bars represent SEM. For all samples: p< 0.05: *, p< 0.01: **, p< 0.001: ***, p< 0.0001: ****
