# Supplementary material for: The Caspase-Activated DNase drives inflammation and contributes to defense against viral infection
Source: Cell Death Differ. 2024 Jun 7;31(7):924–37. doi: 10.1038/s41418-024-01320-7 (PMC11239672; doi:10.1038/s41418-024-01320-7)

**Supplementary Figure S1**

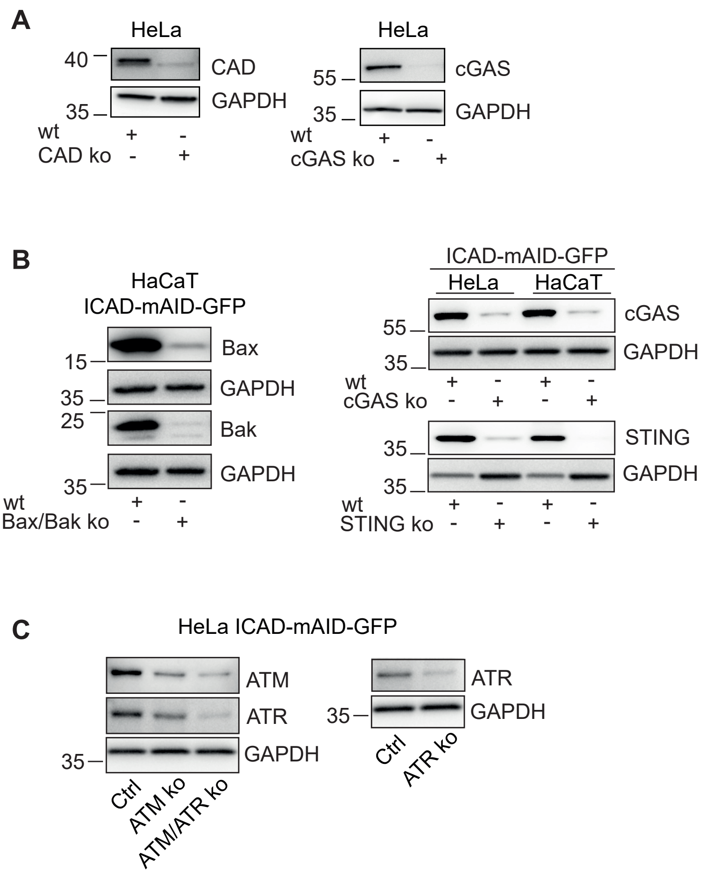

**Supplementary Figure S2**

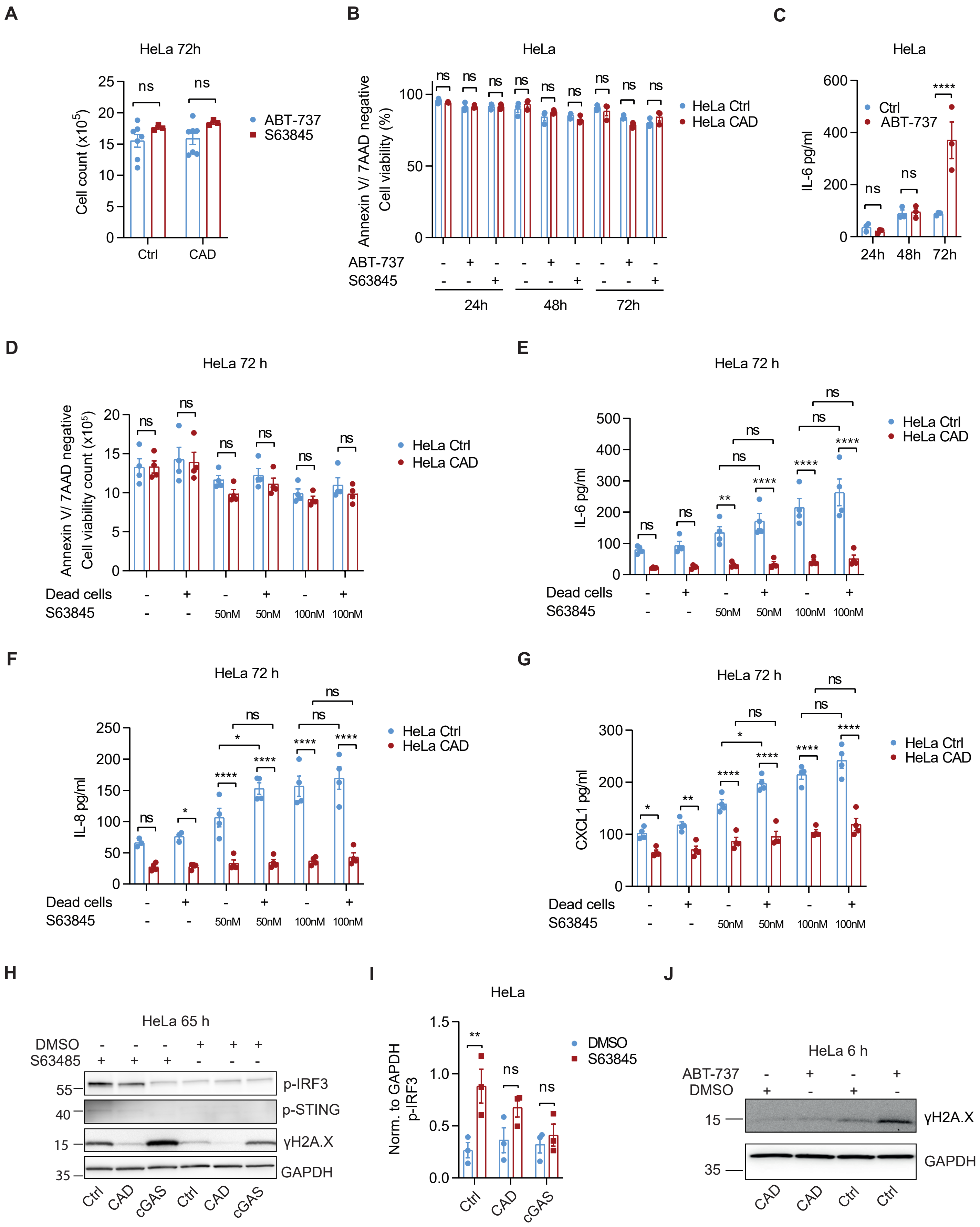

## Supplementary Figure S3

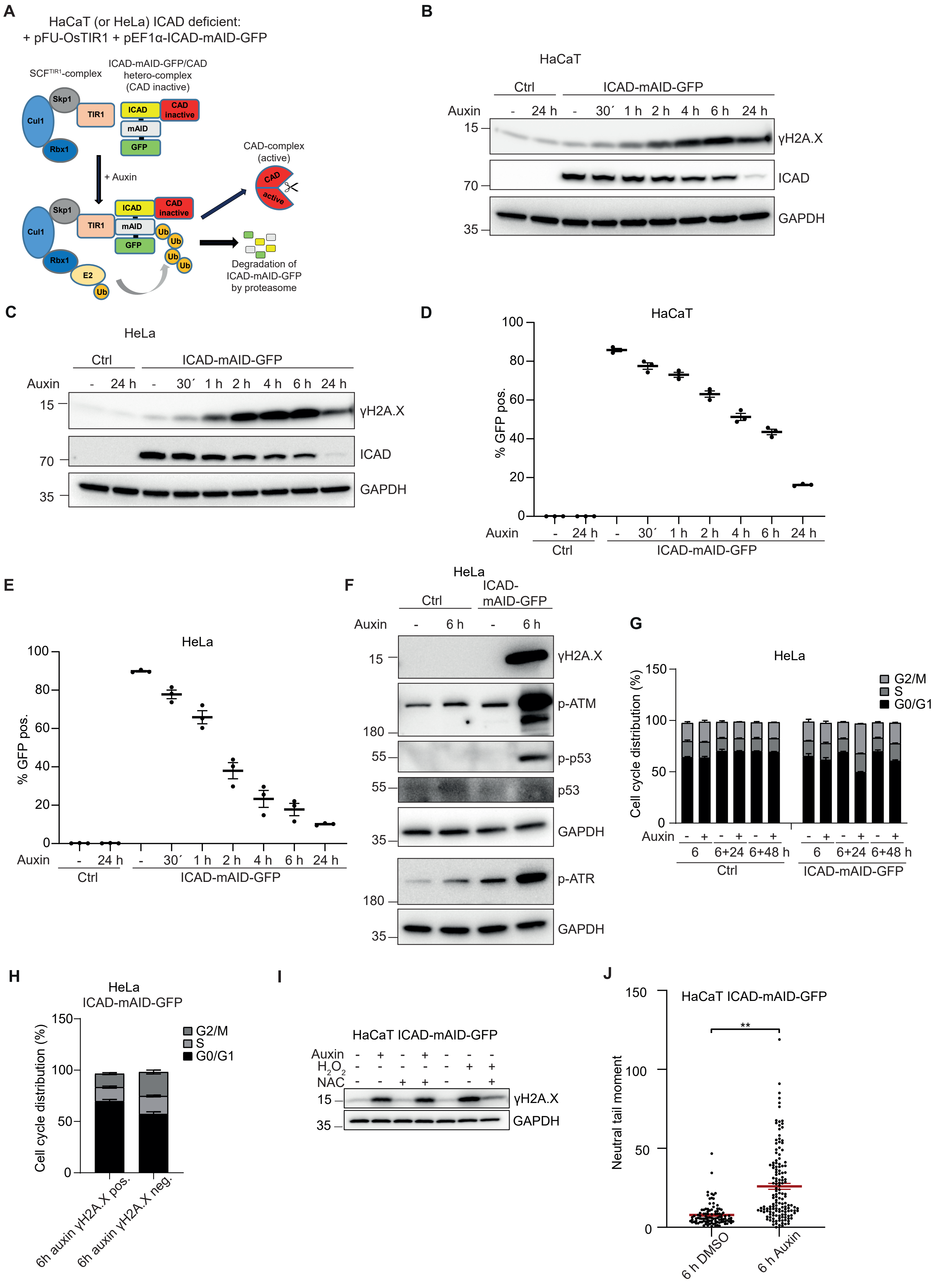

**Supplementary Figure S4**

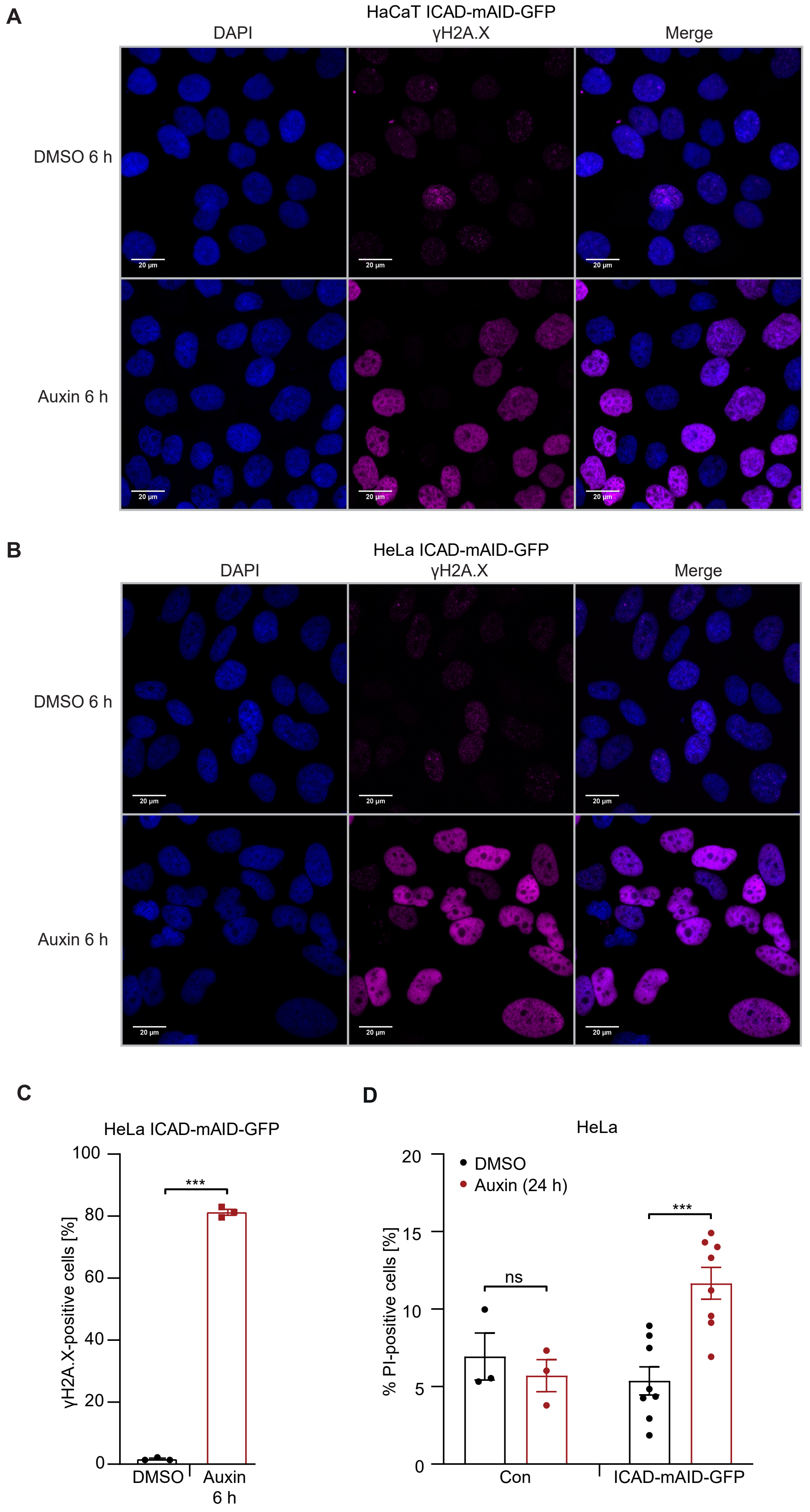

## Supplementary Figure S5

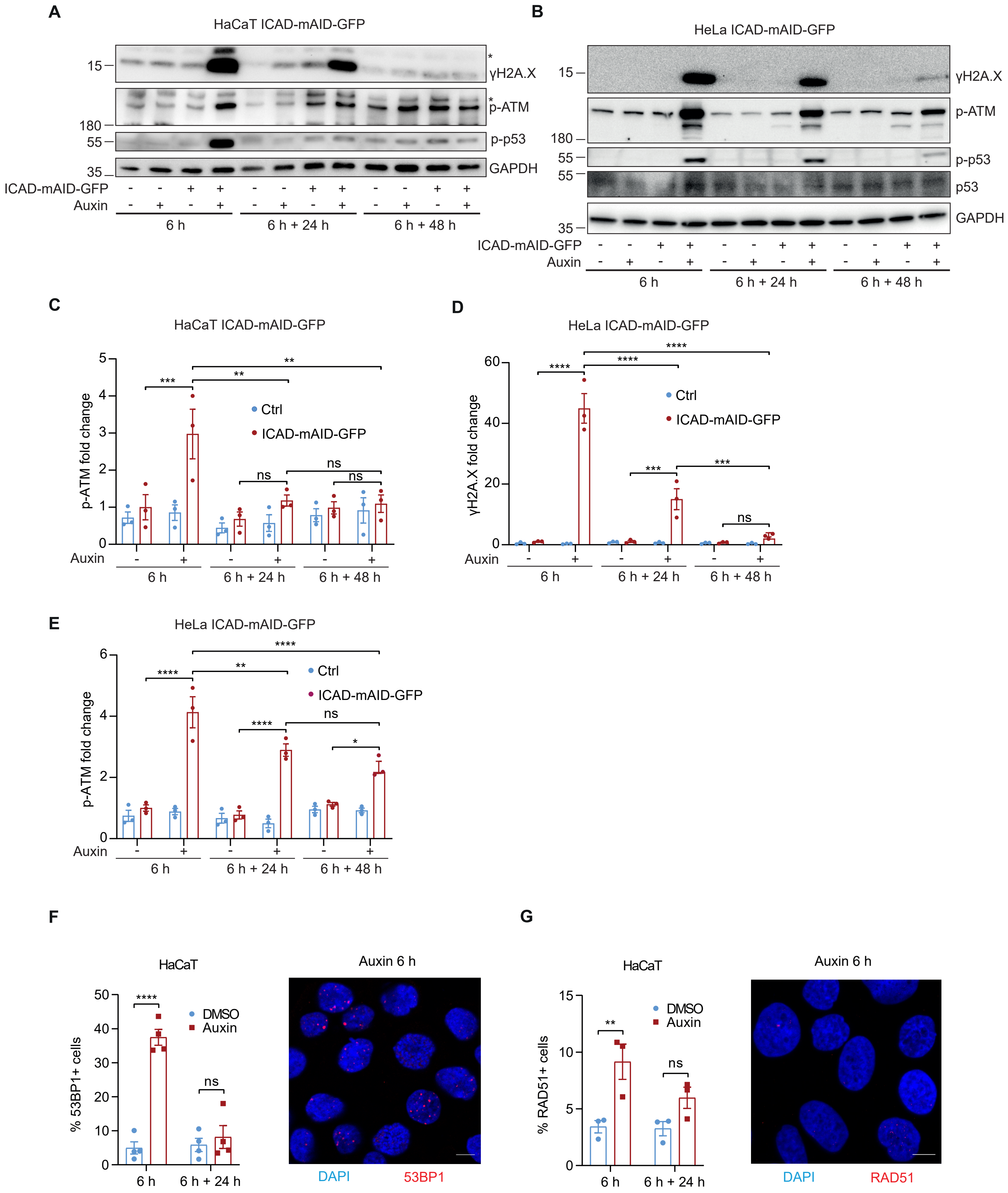

# Supplementary Figure S6

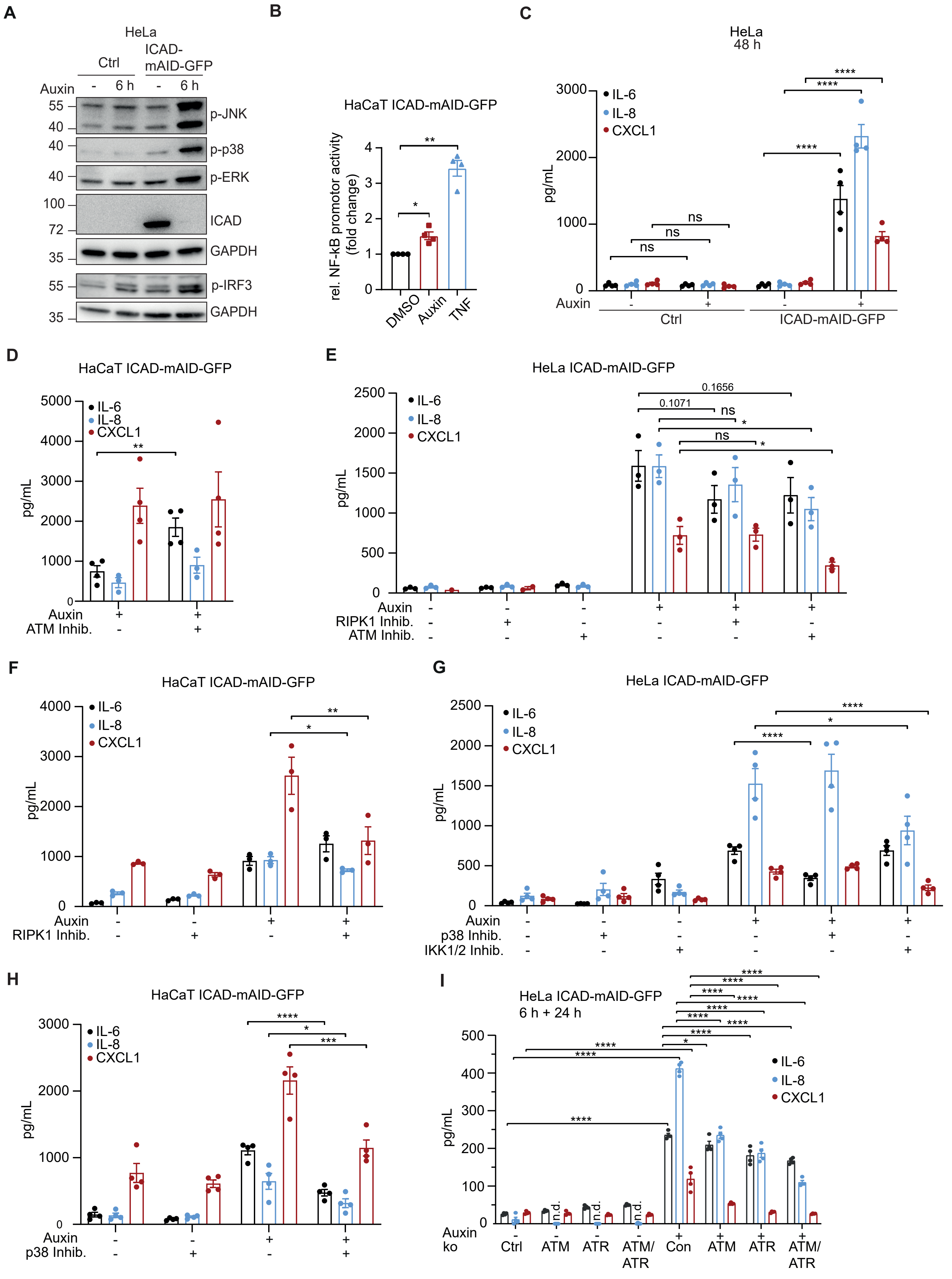

## Supplementary Figure S7

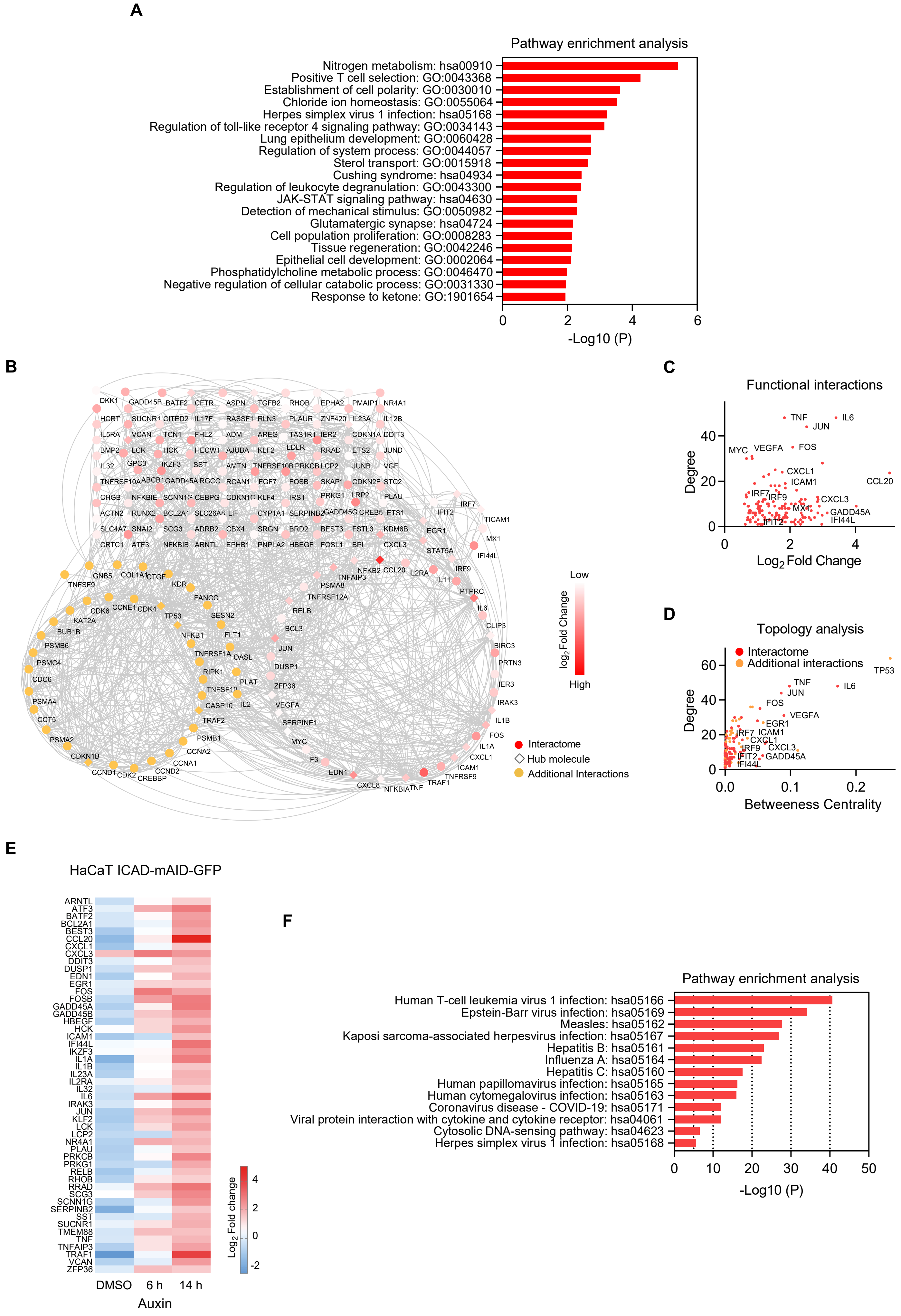

## Supplementary Figure S8

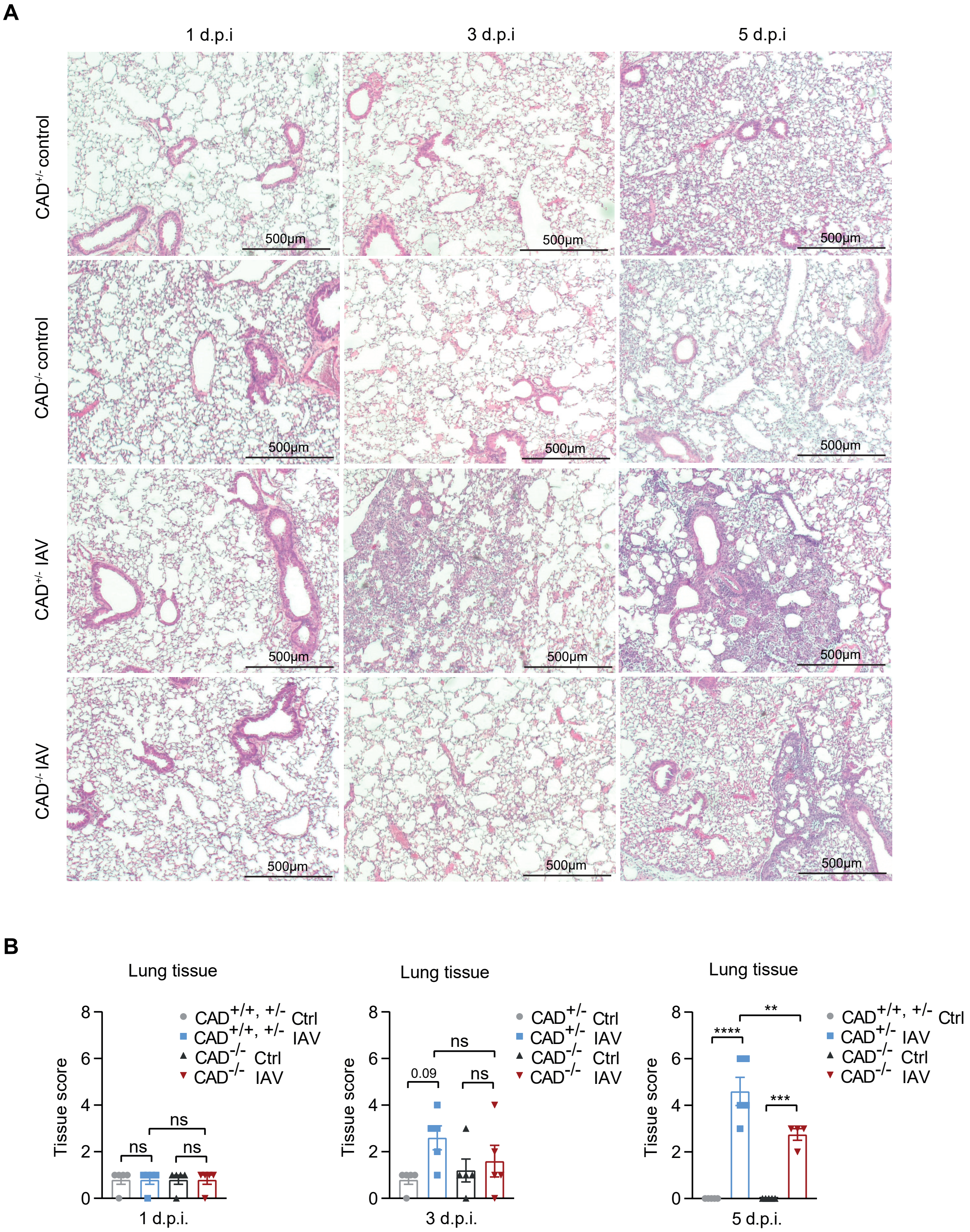

Supplement: Supplementary file 2 — Supplementary figures [file 41418_2024_1320_MOESM2_ESM.pdf]
