# Supplementary material for: The Caspase-Activated DNase drives inflammation and contributes to defense against viral infection
Source: Cell Death Differ. 2024 Jun 7;31(7):924–37. doi: 10.1038/s41418-024-01320-7 (PMC11239672; doi:10.1038/s41418-024-01320-7)

Fig. 1B uncropped WB

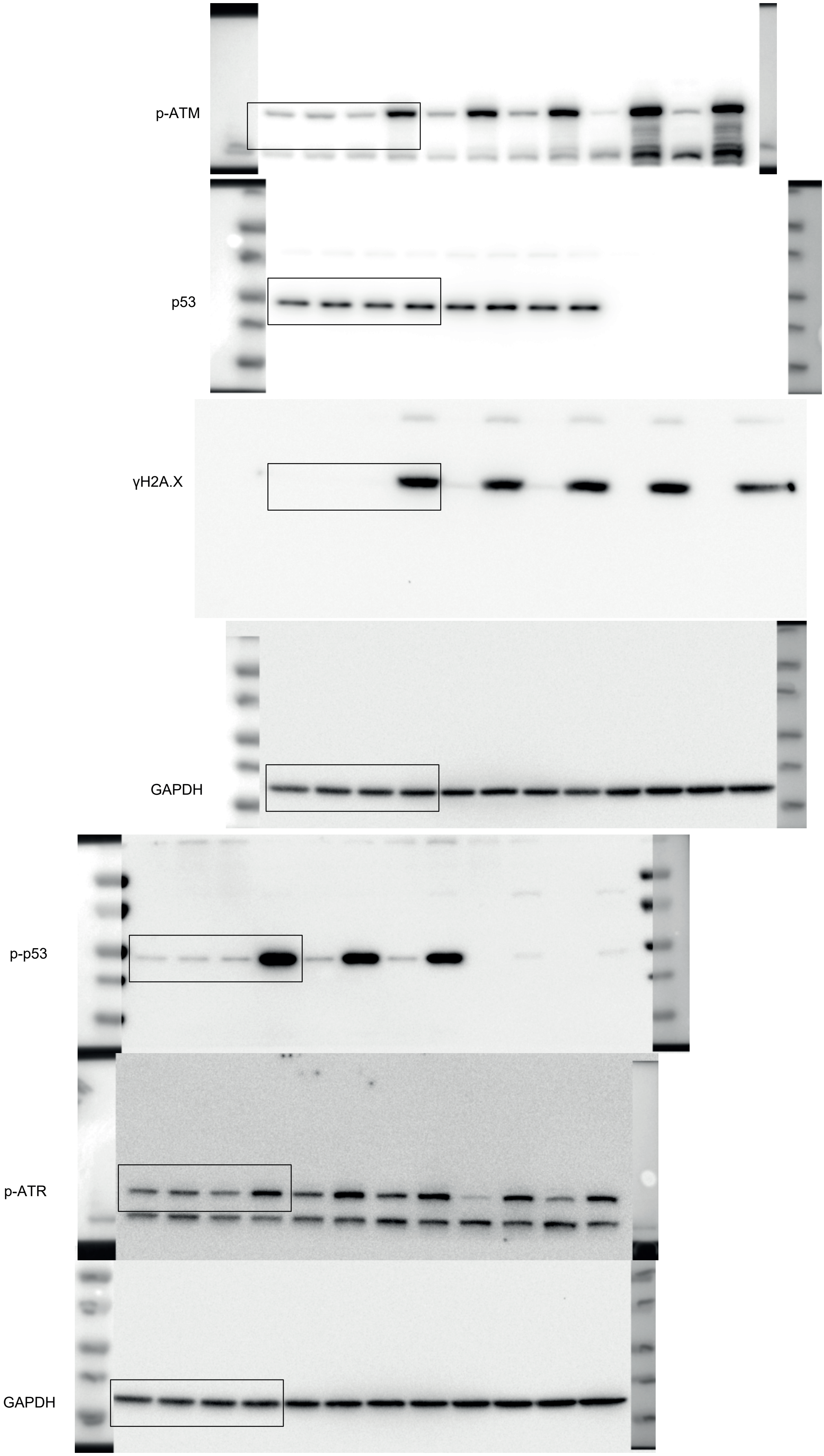

Fig. 2A uncropped WB

p-JNK

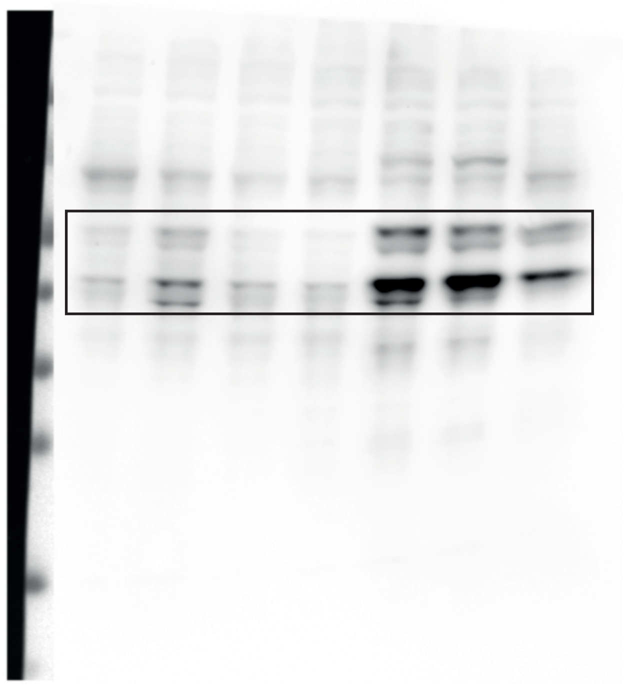

GAPDH

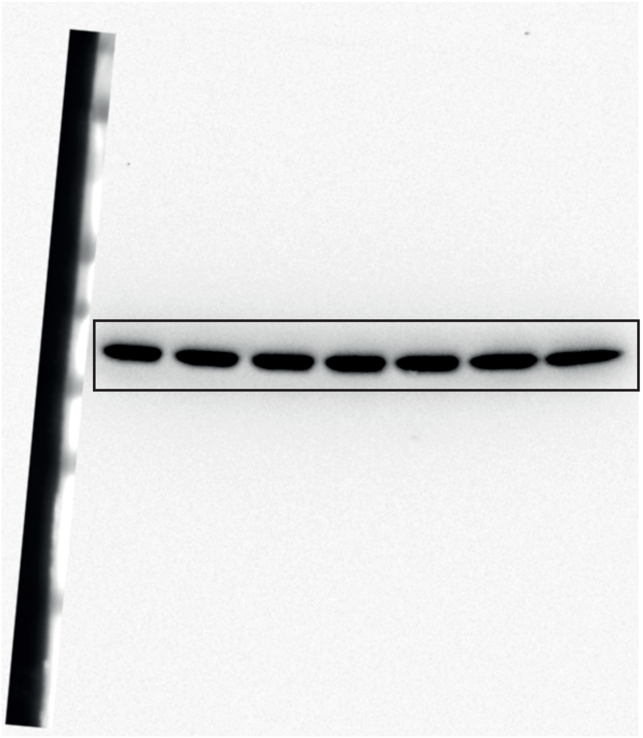

p-p38

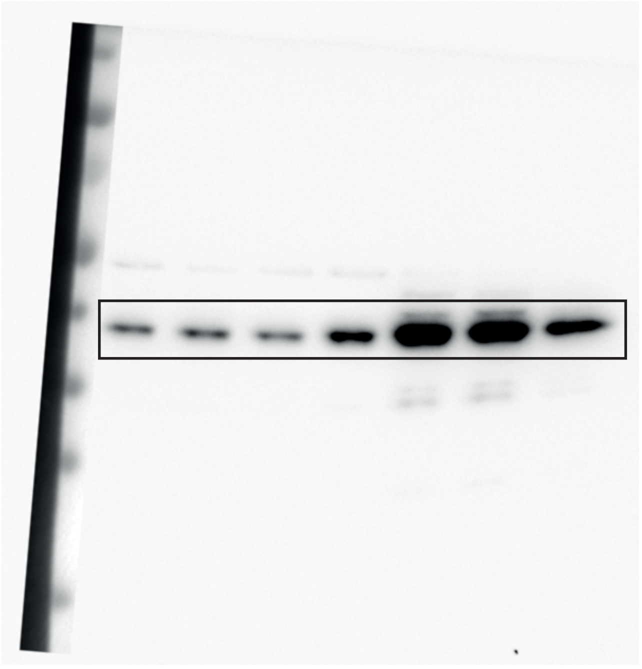

p-IRF3

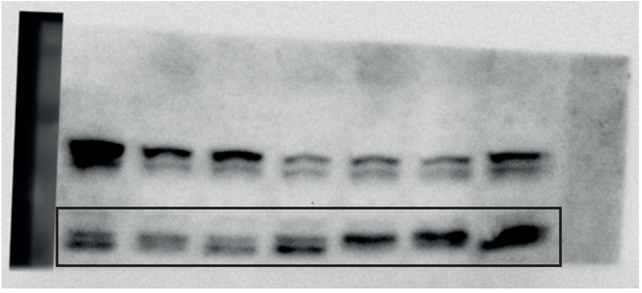

Fig. 2B uncropped WB

p-p65

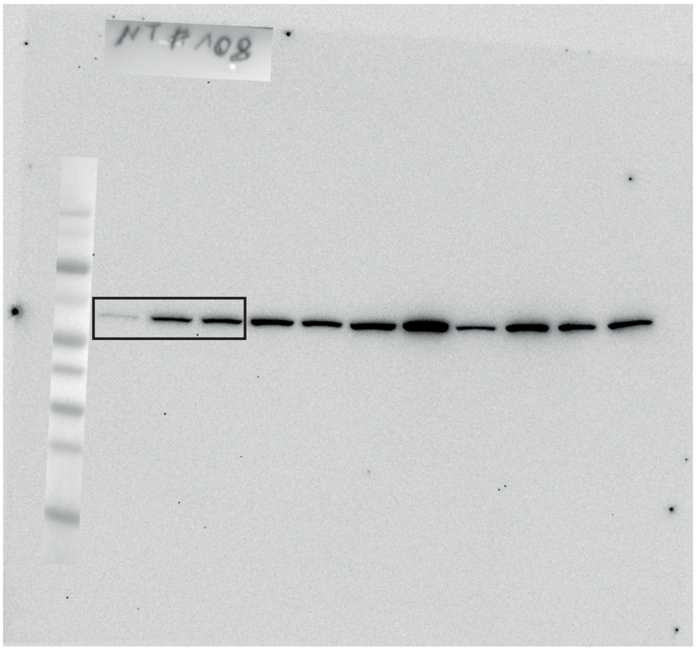

GAPDH

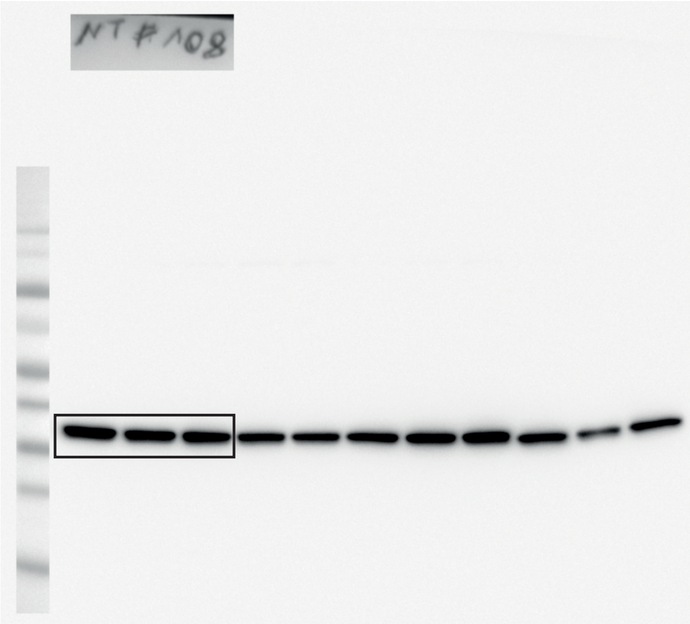

NF-κB p100/p52

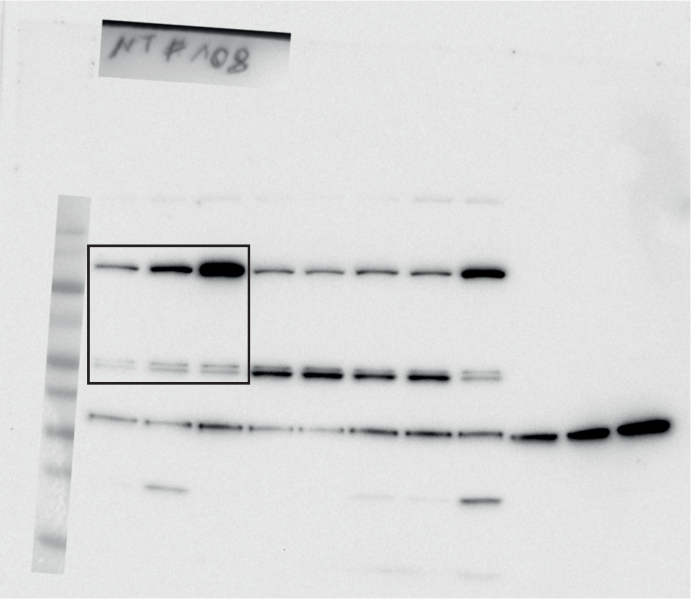

γH2A.X

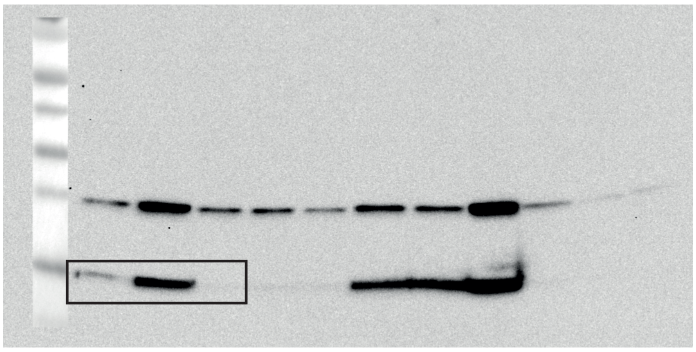

Fig. 2C ucropped WB

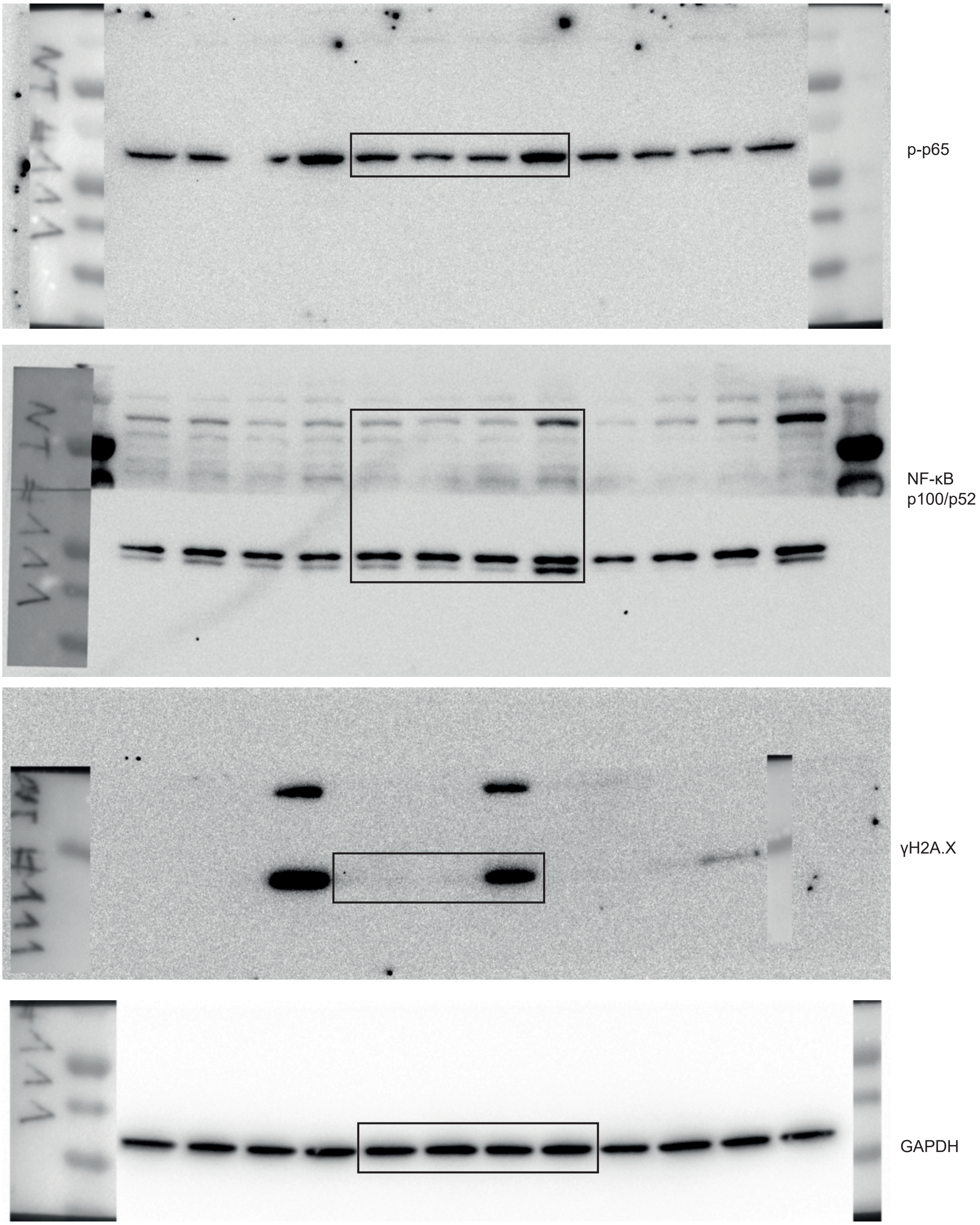

Fig.3E uncropped WB

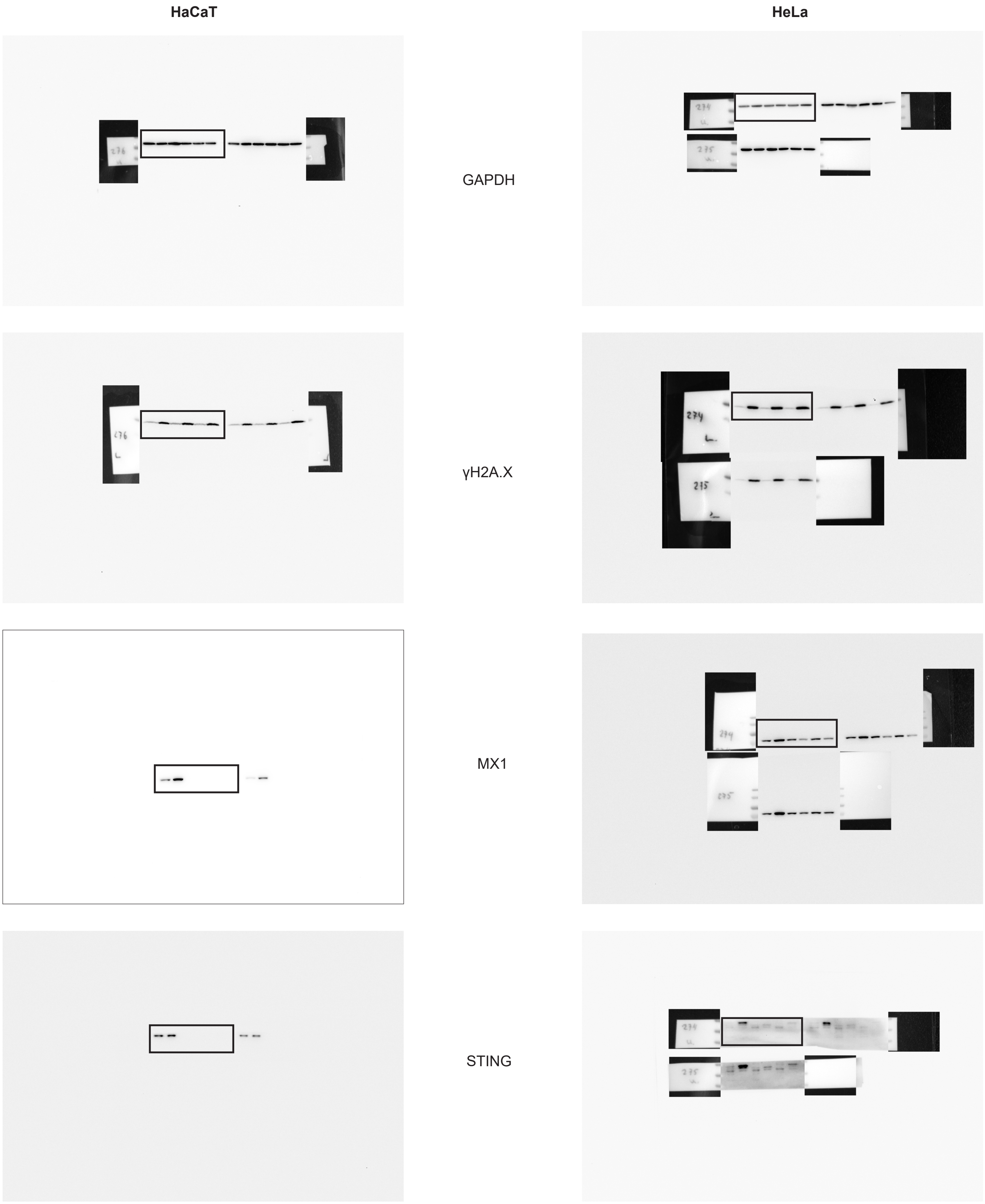

Fig. S1A uncropped WB

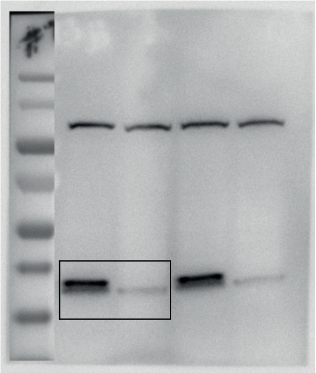

CAD

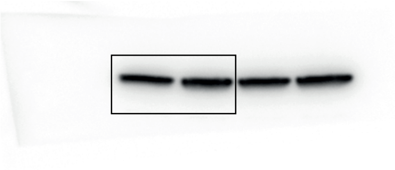

GAPDH

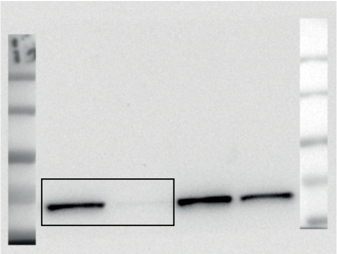

cGAS

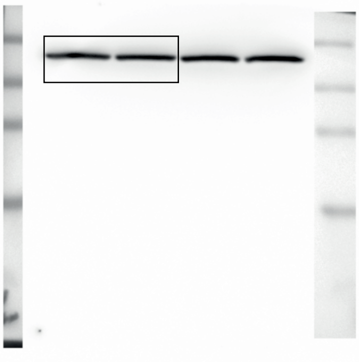

GAPDH

Fig. S1B uncropped WB

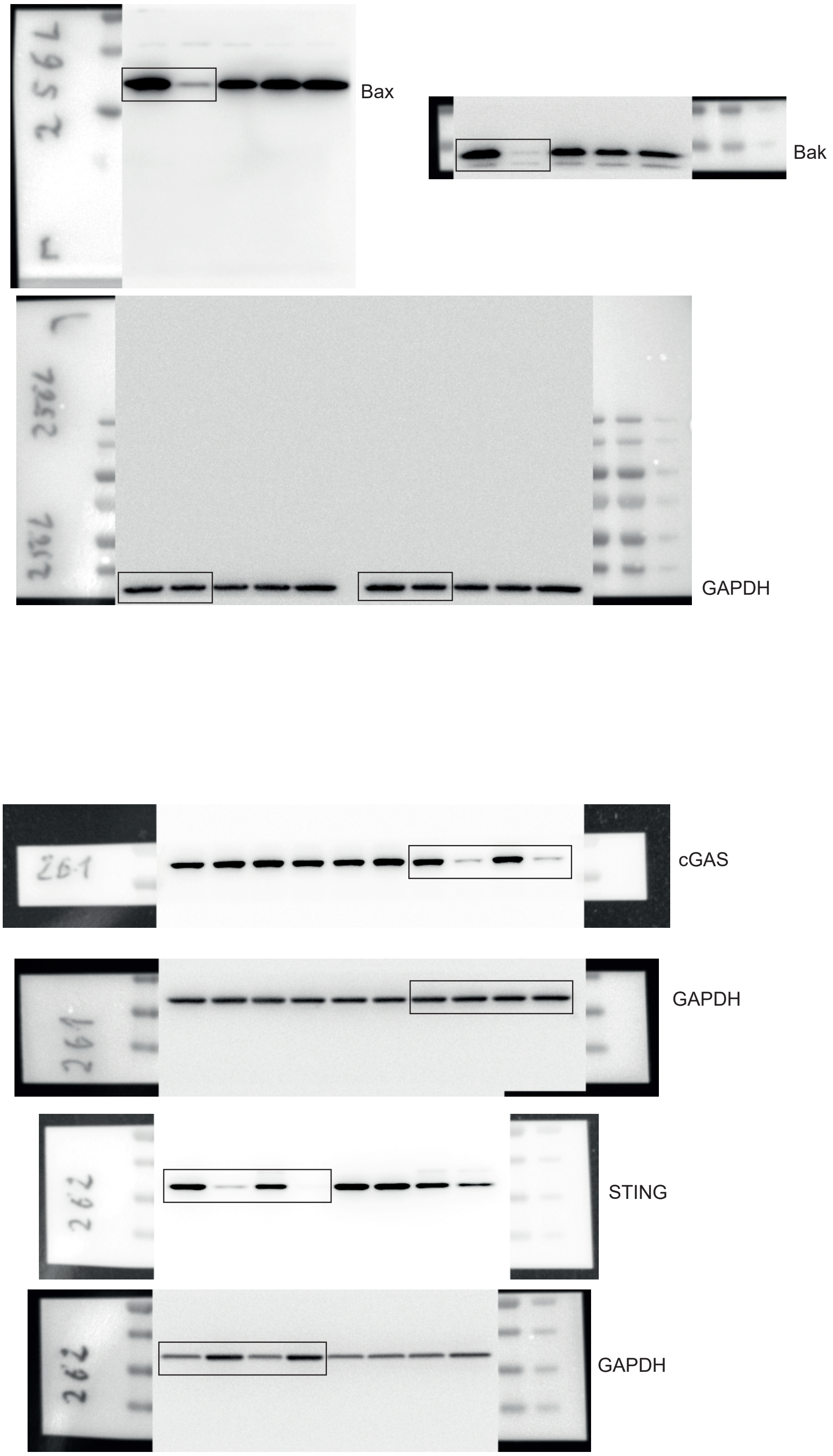

Fig. S1C uncropped WB

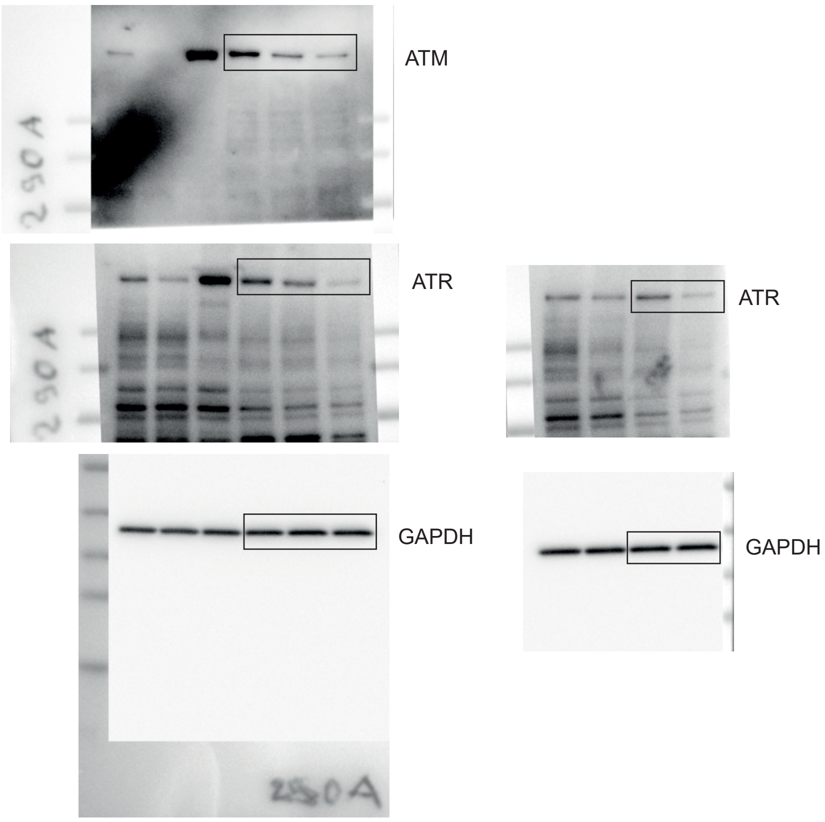

Fig. S2H uncropped WB

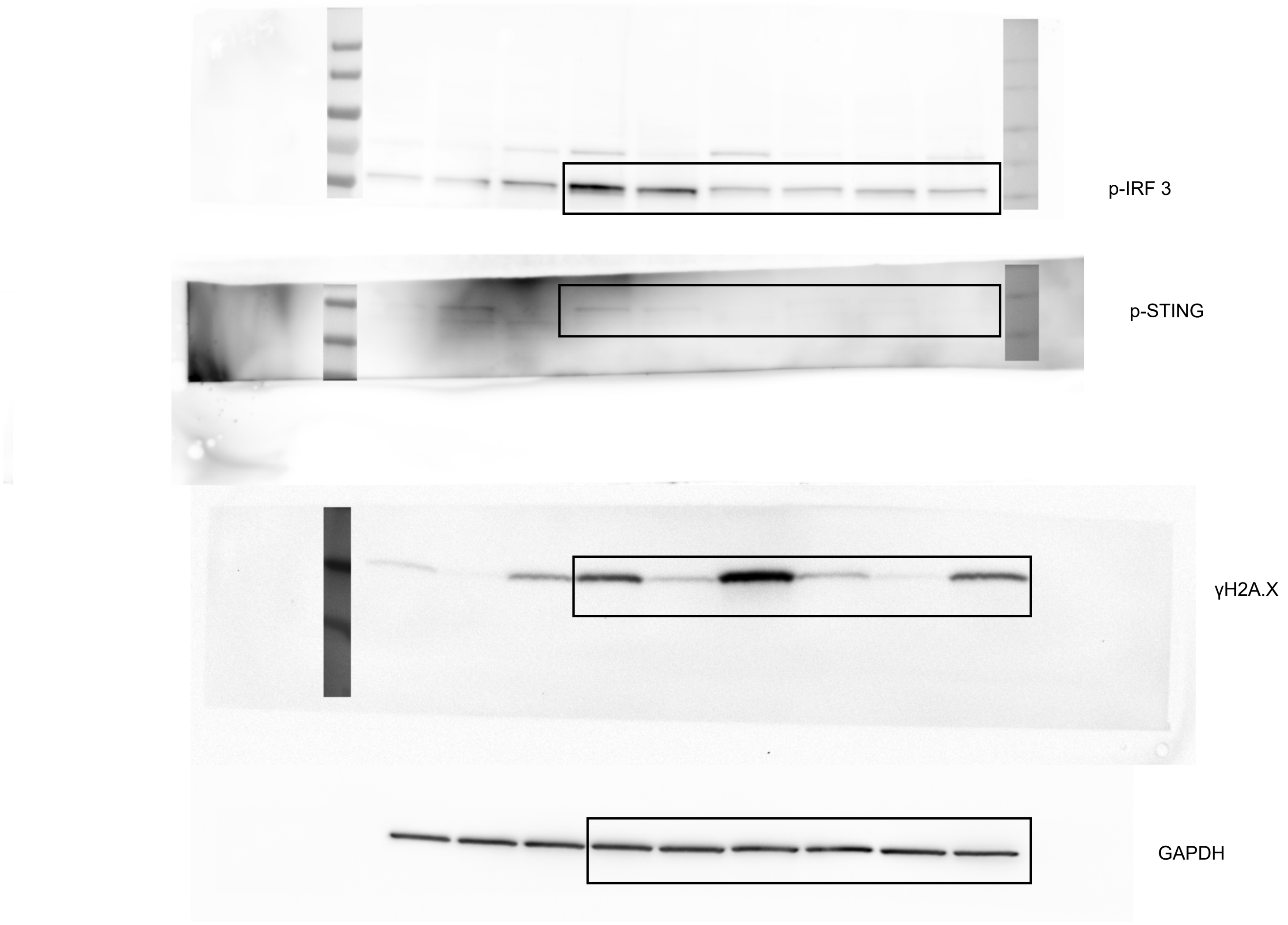

Fig. S2J uncropped WB

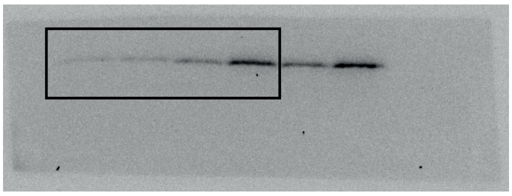

γH2A.X

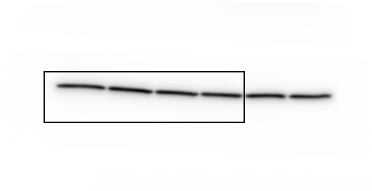

GAPDH

Fig S3B-C uncropped WB

γH2A.X

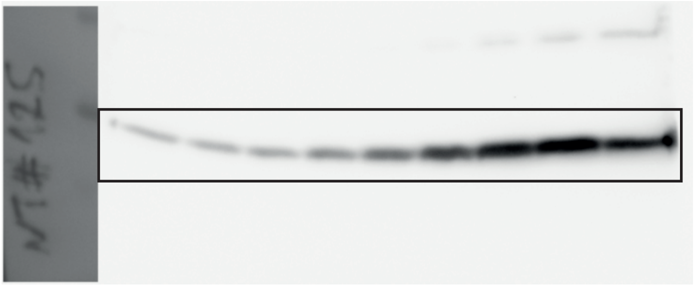

i-CAD

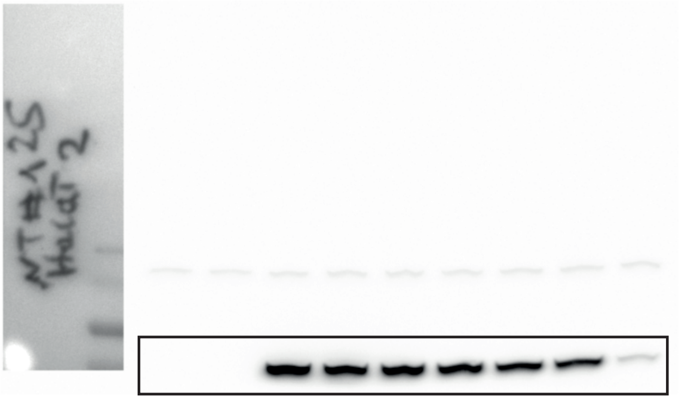

GAPDH

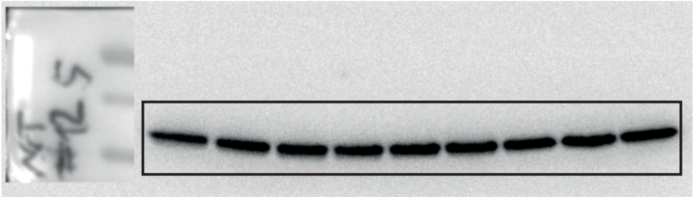

HaCaT

γH2A.X

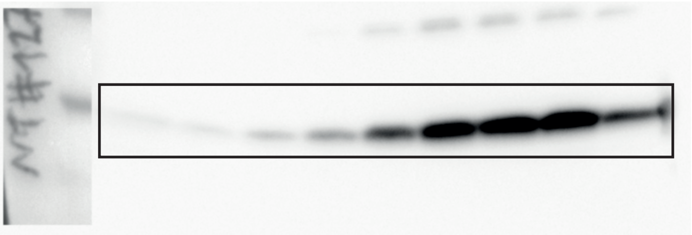

i-CAD

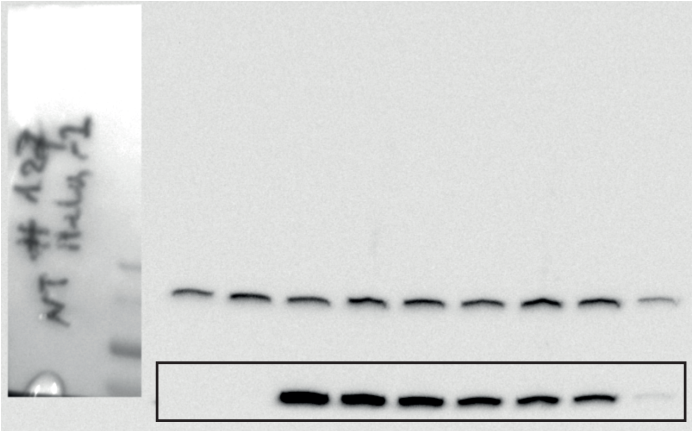

GAPDH

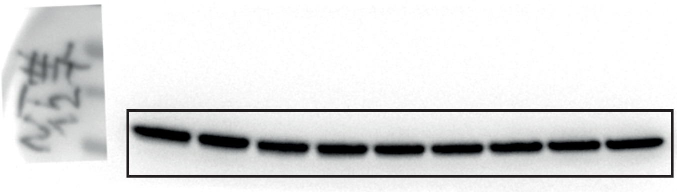

HeLa

Fig. S3F uncropped WB

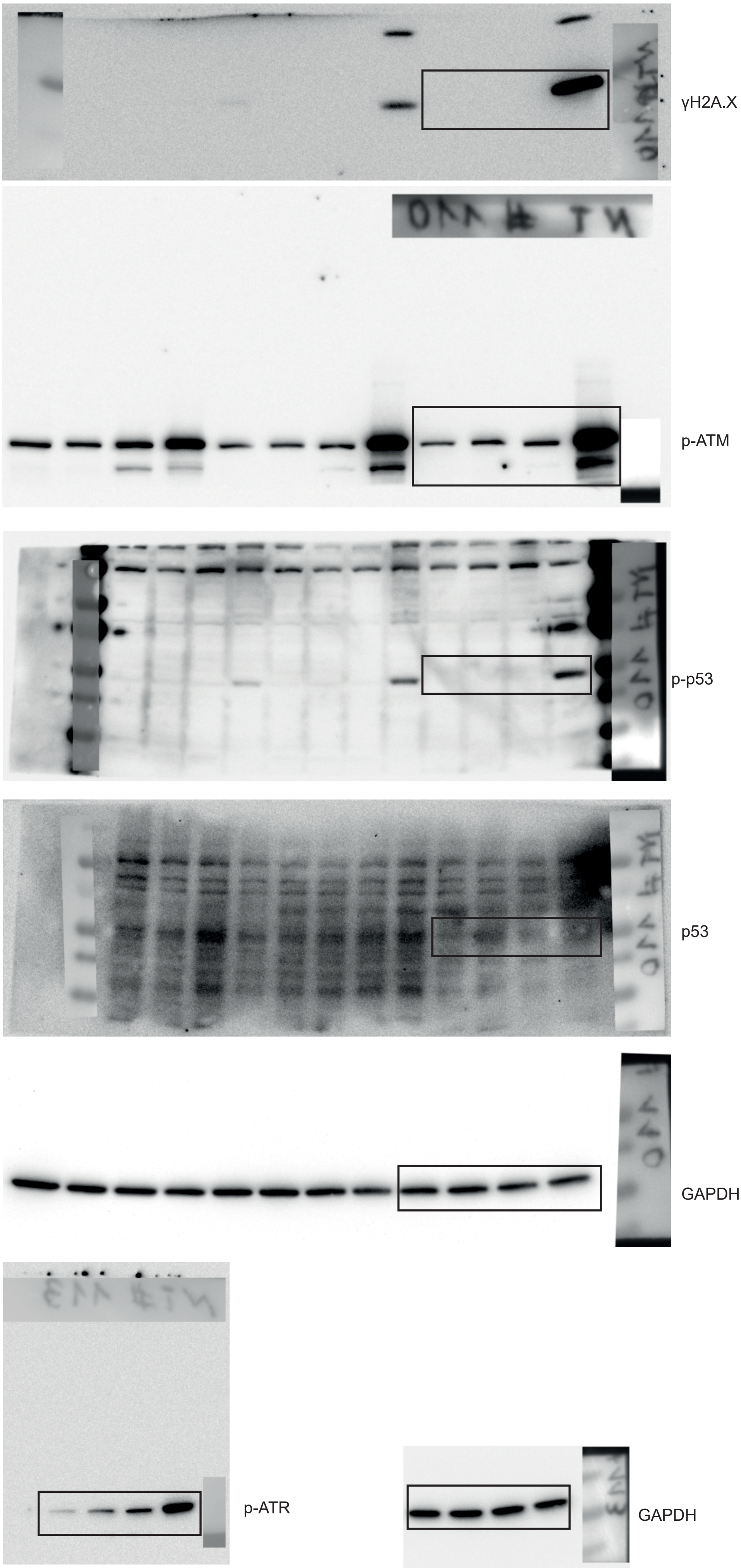

Fig. S3I uncropped WB

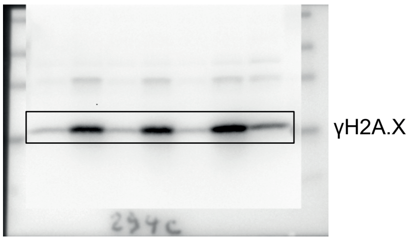

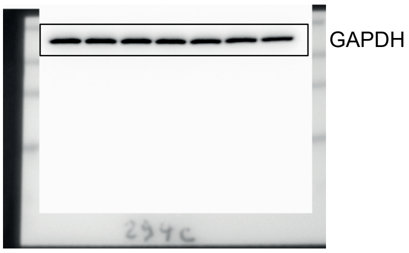

Fig. S5A uncropped WB

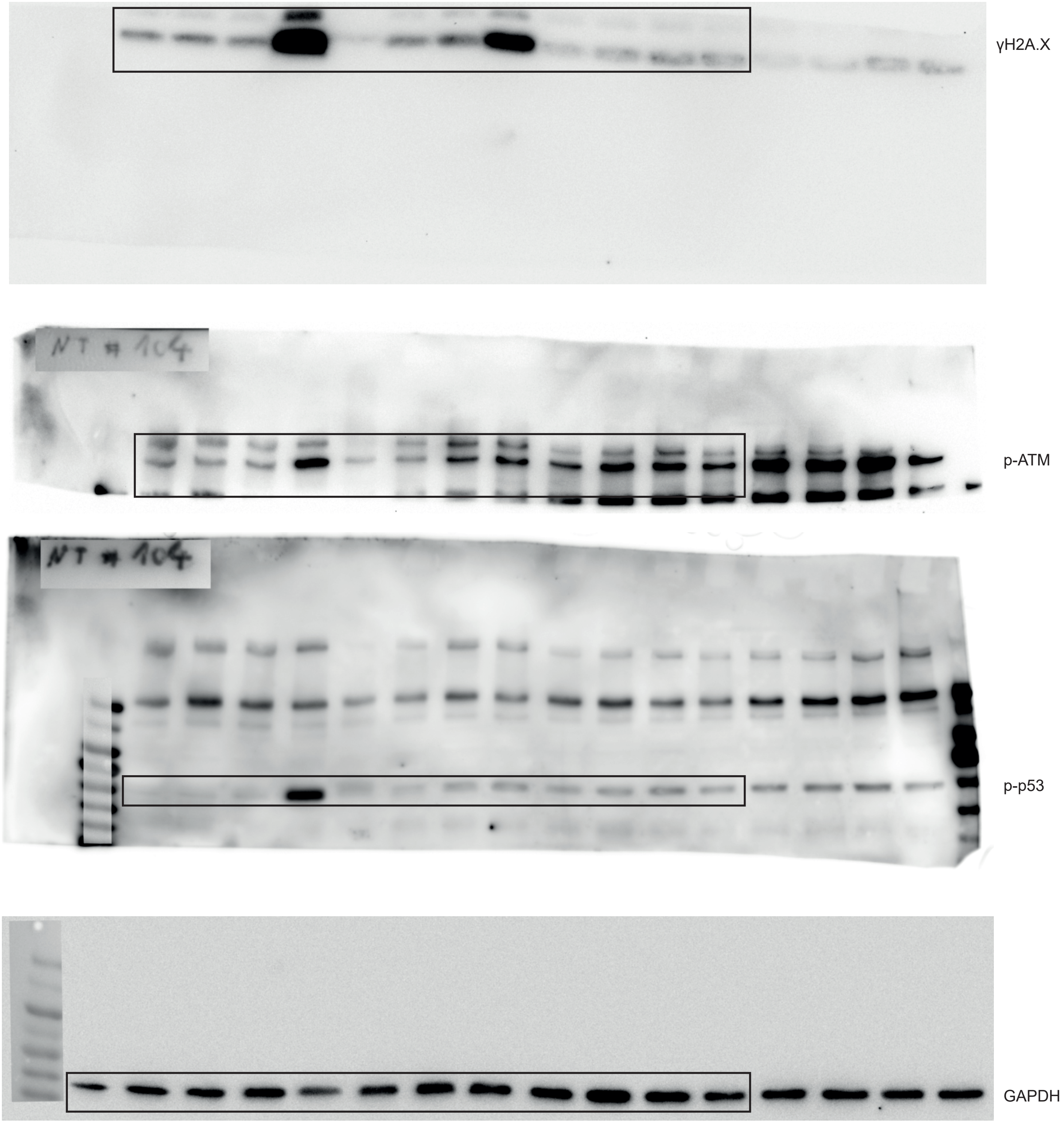

Fig. S5B uncropped WB

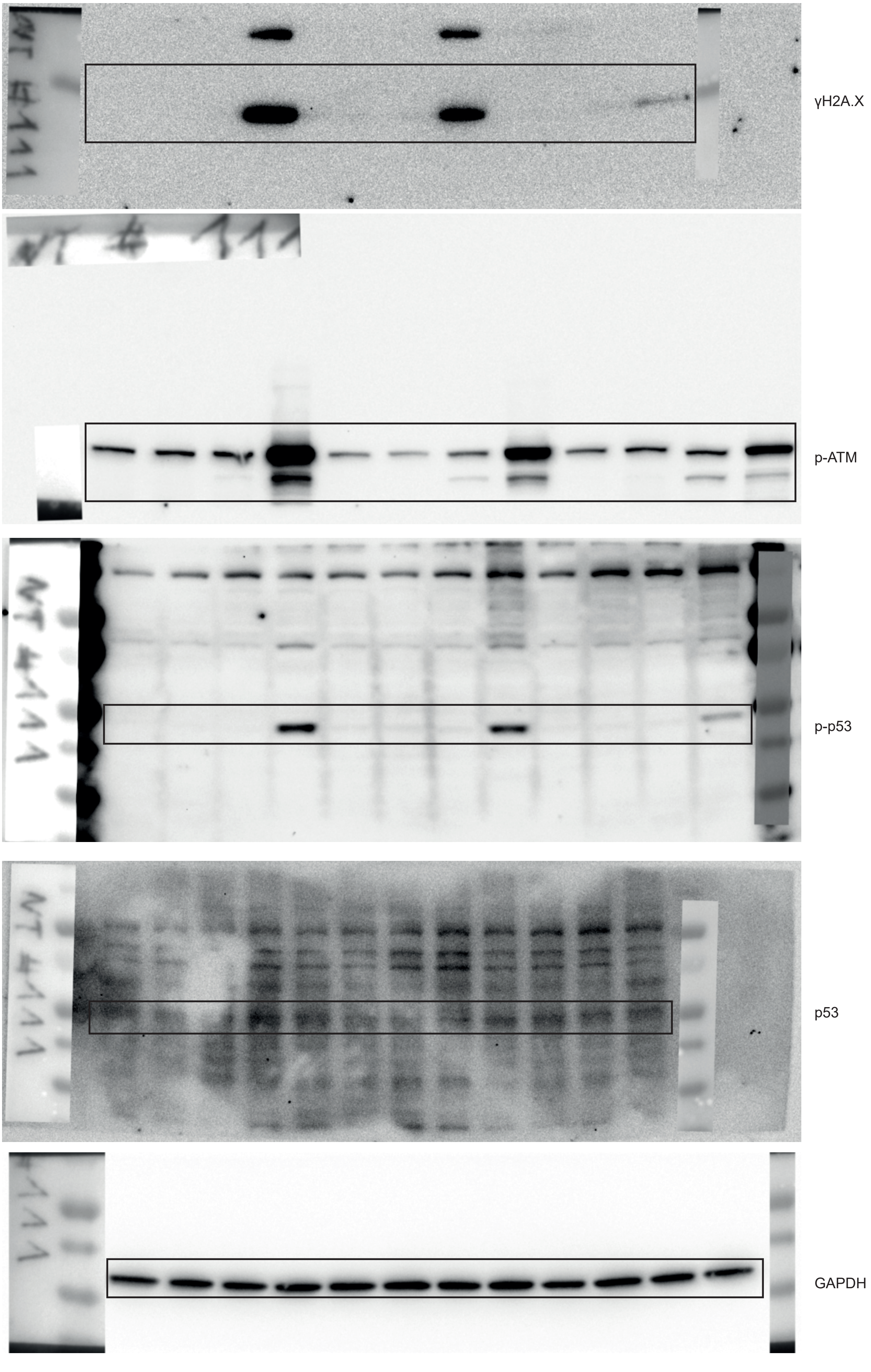

Fig. S6A uncropped WB

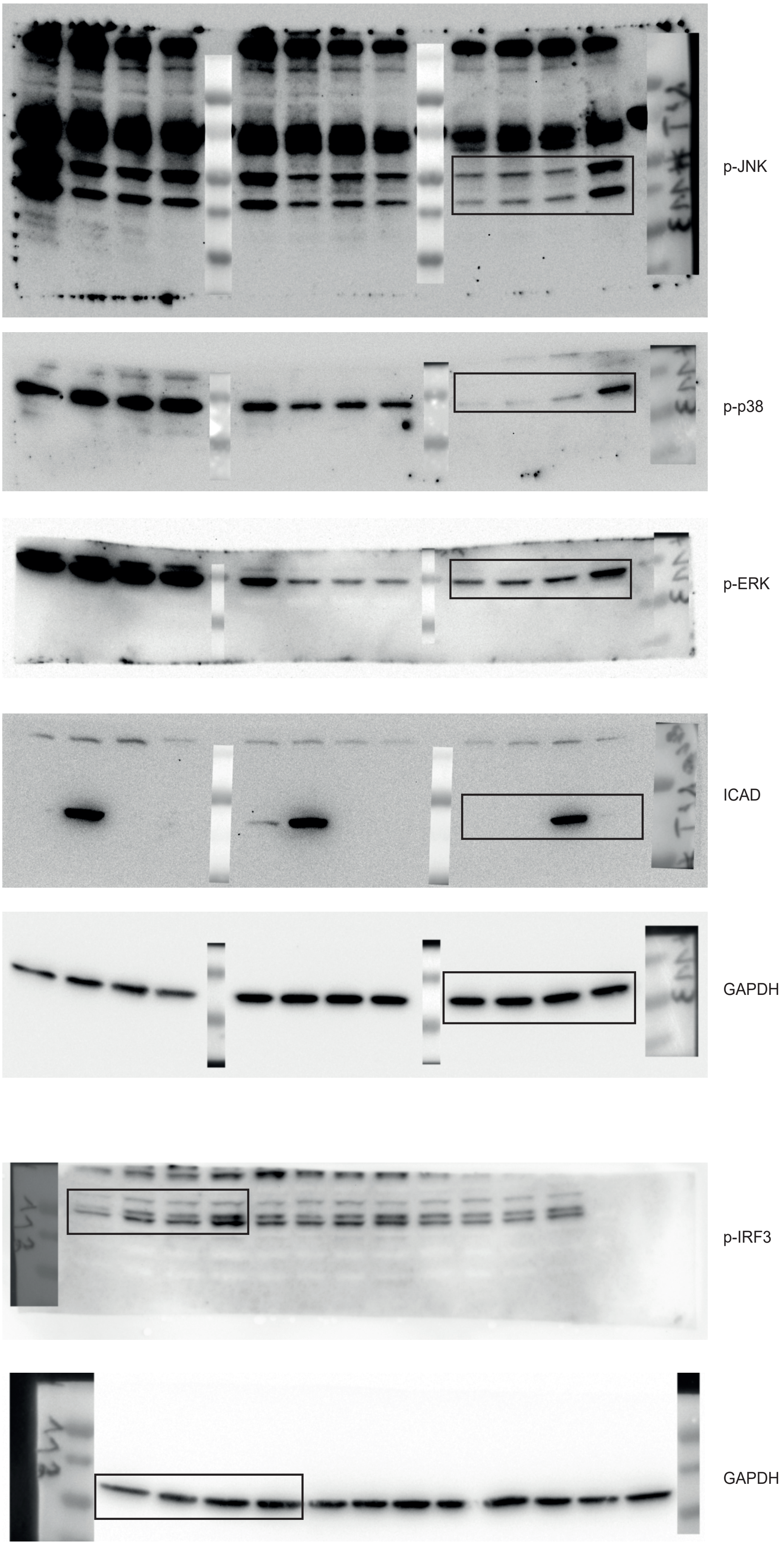

Supplement: Supplementary file 3 — Uncropped western blot figures [file 41418_2024_1320_MOESM3_ESM.pdf]
